# Uncovering the molecular targets of phytocannabinoids: mechanistic insights from inverse molecular docking fingerprint approaches

**DOI:** 10.3389/fphar.2025.1611461

**Published:** 2025-06-27

**Authors:** Vid Ravnik, Marko Jukič, Veronika Furlan, Uroš Maver, Jan Rožanc, Urban Bren

**Affiliations:** ^1^ Faculty of Chemistry and Chemical Engineering, University of Maribor, Maribor, Slovenia; ^2^ The Faculty of Mathematics, Natural Sciences and Information Technologies, University of Primorska, Koper, Slovenia; ^3^ Institute of Biomedical Sciences, Faculty of Medicine, University of Maribor, Maribor, Slovenia; ^4^ Department of Pharmacology, Faculty of Medicine, University of Maribor, Maribor, Slovenia; ^5^ Institute for Environmental Protection and Sensors, Maribor, Slovenia

**Keywords:** cannabinoids, Cannabis sativa, inverse molecular docking, inverse molecular docking fingerprints, mode of action, virtual screening, drug design

## Abstract

**Introduction:**

Among diverse chemical profile of *Cannabis sativa L.*, over 100 phytocannabinoids have been identified. The major cannabinoids Δ -9-THC and CBD are well-studied, with approved palliative and therapeutic applications such as appetite stimulation, antiemetic therapy, pain management and epilepsy treatment. However, Δ -9-THC’s psychotropic effects limit its broader use. Minor cannabinoids exhibit therapeutic promise for a variety of conditions, potentially offering therapeutic potential without the adverse effects of Δ -9-THC.

**Methods:**

We explored 14 cannabinoids with an inverse molecular docking approach, docking each cannabinoid into >50000 human protein structures from the ProBiS-Dock database. We validated our inverse molecular docking protocol using retrospective metrics (ROC AUC, BEDROC, RIE, enrichment factors, total gain). We apply the novel inverse molecular docking fingerprint method to better analyze the binding patterns of different cannabinoids and extend the methodology to include hierarchical clustering of fingerprints.

**Results:**

Our analysis of the inverse molecular docking results identified high scoring targets with potential as novel protein targets for minor cannabinoids, the majority associated with cancer, while others have connections with neurological disorders and inflammation. We highlighted GTPase KRas and hematopoietic cell kinase (HCK) as very promising potential targets due to favorable docking scores with almost all investigated cannabinoids. We also find multiple matrix metalloproteinases among the top targets, suggesting possible novel therapeutic opportunities in rheumatic diseases. An analysis of inverse molecular docking fingerprints shows similar binding patterns for cannabinoids with similar structures, minor structural differences still suffice to change the affinity to specific targets. Hierarchical clustering of inverse molecular docking fingerprints revealed two main clusters in protein binding pattern similarity, the first encompassing THC-class and similar cannabinoids, as well as CBL-class cannabinoids, while the second contained CBD, CBC, and CBG-class cannabinoids. Notably, CBL-class cannabinoids exhibited binding patterns more similar to THC-class cannabinoids than their CBC-class precursors, possibly offering potential therapeutic benefits akin to THC with fewer psychotropic effects.

**Discussion:**

This study highlights the therapeutic potential of minor cannabinoids and identifies their potential novel protein targets. Moreover, we demonstrate the utility of inverse molecular docking fingerprinting with clustering to identify compounds with similar binding patterns as well as identify pharmacophore-related compounds in a structurally agnostic manner, paving the way for future drug discovery and development.

## 1 Introduction

The significant potential for beneficial health effects of *Cannabis* use represents a major research topic. *Cannabis sativa L.* is widely distributed in various environments and has been applied as a source of folk medicines, textile fibers, and as a psychoactive agent for over 6,000 years (Atakan, 2012). *Cannabis* extracts include a large variety of chemical compounds, with over 500 substances already isolated. Over 100 of these compounds represent phytocannabinoids, unique secondary metabolites of *Cannabis* sharing similar structural features (ElSohly and Gul, 2014; Hanuš et al., 2016).

Phytocannabinoids, hereinafter referred to as cannabinoids, include meroterpenoids typical of *Cannabis sativa L.* Their defining structural feature is a resorcinyl core decorated with *para* oriented terpenyl and alkyl groups. The length of the side-chain alkyl group distinguishes between different classes of cannabinoids. The most common cannabinoids fall into the olivetoid class, distinguished by a five-carbon side chain. Less frequent are viridinoids (three-carbon chain), orcinoid (one carbon, rare in *Cannabis*), and cannabinoids with aralkyl side chains (Hanuš et al., 2016). The decarboxylated olivetoid cannabinoids are typically identified by the three-letter abbreviation (e.g., CBD (cannabidiol)), while the acidic version includes an additional “A” (e.g., CBDA (cannabidiolic acid)). For viridinoids, a “V” is added (e.g., CBDV (cannabidivarin)), while acidic viridinoids are designated with “VA” (e.g., CBDVA (cannabidivarinic acid)).

**FIGURE 1:**
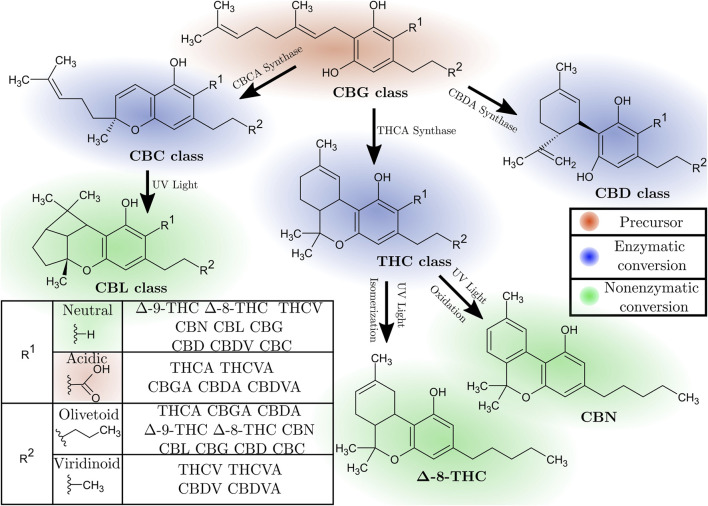
Overview of the major classes of cannabinoid compounds and the three main pathways of their biosynthesis. CBGA and CBGVA serve as common precursors, with neutral cannabinoids primarily forming through non-enzymatic decarboxylation. The table outlines the R groups of cannabinoids used in this study: ${\text{R}}^{1}$ distinguishes neutral from acidic cannabinoids, and ${\text{R}}^{2}$ differentiates olivetoid from viridinoid cannabinoids. Note that for CBL and CBC-class cannabinoids, both enantiomers occur naturally.

Cannabinoid biosynthesis *in planta* begins with cannabigerolic acid (CBGA), the common cannabinoid precursor of other (olivetoid) cannabinoids. CBGA undergoes oxidative cyclase activity via three distinct pathways with specific enzymes, to form cannabichromenic acid (CBCA), cannabidiolic acid (CBDA), or tetrahydrocannabinolic acid (THCA), see Figure 1. Neutral cannabinoids, such as cannabigerol (CBG), cannabichromene (CBC), cannabidiol (CBD), or Δ -9-tetrahydrocannabinol ( Δ -9-THC), are primarily formed through the non-enzymatic decarboxylation of their carboxylated counterparts. Poor oxidative stability of Δ -9-THC can result in conversion to cannabinol (CBN), or in isomerization to Δ -8-THC (or Δ -10-THC). Cannabicyclolic acid (CBLA) and cannabicyclol (CBL) result from the UV-induced cycloaddition of CBCA and CBC, respectively. (Pellati et al., 2018; Hanuš et al., 2016) (Figure 1).

Research into cannabinoid ( Δ -9-THC) physiological and pharmacological effects began with the discovery of cannabinoid receptors, and their endogenous ligands endocannabinoids. Derived from long-chain polyunsaturated fatty acids, endocannabinoids anandamide (AEA) and 2-arachidonoylglycerol (2-AG) represent lipid mediators that replicate many of the pharmacological effects of Δ -9-THC (Maccarrone and Finazzi-Agro, 2003). The endocannabinoid system (ECS) is the network of interactions of cannabinoid receptors, endocannabinoids, and enzymes that generate, transform, and degrade them. Cannabinoid receptor 1 (CB1) is found primarily in the central nervous system, while CB2 is present mostly in immune-related tissues (Lu and Mackie, 2021; Finn et al., 2021). Cannabinoids also interact with G-protein-coupled receptors (e.g., GPR55 and GPR18), transient receptor potential (TRP) channels, and peroxisome proliferator-activated receptors (PPARs). TRP channels represent ionotropic channels that are primarily activated by physical, thermal, and electrochemical stimuli. PPARs are nuclear receptors, which function as ligand-inducible transcription factors (Atakan, 2012; Alexander, 2016; Fraguas-Sánchez and Torres-Suárez, 2018; Walsh et al., 2021). Studies have predominantly focused on major cannabinoids Δ -9-THC and CBD, while the less abundant minor cannabinoids have remained relatively understudied. Minor cannabinoid pharmacology is not yet fully understood, however, evidence demonstrates they act as agonists or antagonists on multiple targets, such as cannabinoid receptors, and the above-listed targets (Walsh et al., 2021). The currently known mechanisms of action and therapeutic potential of different minor cannabinoids were reviewed by Ref. Walsh et al. (2021).

Besides plant-derived phytocannabinoids and endogenous endocannabinoids, synthetic cannabinoids have also been developed and studied. They are often developed as modulators of CB receptors, possessing stronger biological activity than Δ -9-THC. However, the anticipated stronger psychoactive effects and the perception of lower risk of synthetic cannabinoid application can lead to abuse and represents a rising health concern (Mills et al., 2015; Alves et al., 2020; Roque-Bravo et al., 2023).

Major cannabinoids have proven successful in clinical practice, for example, Δ -9-THC (Dronabinol, sold under Syndros^®^, Marinol^®^), is approved in the U.S. as an appetite stimulant for AIDS patients and as an antiemetic alongside chemotherapy. However, the medicinal use of Δ -9-THC is limited by its psychotropic effects (Kaufmann et al., 2010). Moreover, pure, plant derived CBD (Epidiolex^®^) is approved for use in patients with resistant epileptic syndromes (Fraguas-Sánchez and Torres-Suárez, 2018). Besides applications of pure cannabinoids, a large body of research focuses on therapeutic use of cannabinoid enriched extracts of the *Cannabis* plant (Fadda et al., 2004; Lakhan and Rowland, 2009; Hussain et al., 2015; Schrot and Hubbard, 2016; Schonhofen et al., 2018; Zagórska-Dziok et al., 2021). According to several studies, the therapeutic potential of cannabinoids is enhanced when they are alongside other bioactive compounds in *Cannabis*, often named the “entourage effect” (Worth, 2019; André et al., 2024). The term entourage effect refers to the synergistic effects of several compounds from *Cannabis*, namely, cannabinoids, terpenes, and flavonoids, leading to the enhanced therapeutic effects when compared to the effects of individual compounds (André et al., 2024). Two different types of entourage effects have been reported for cannabis-derived compounds, namely, intra-entourage effect, which refers to the interactions among cannabinoids or terpenes, and inter-entourage effect, involving to interactions between cannabinoids and terpenes (Koltai and Namdar, 2020).

As an example of intra-entourage effects, Nabiximols (Sativex^®^), a roughly 1:1 formulation of Δ -9-THC and CBD, has been approved in Canada, Mexico, and several European countries for treating spasticity associated with multiple sclerosis (Fraguas-Sánchez and Torres-Suárez, 2018). Although heavy chronic and recreational use of Nabiximols (Sativex^®^) can lead to addiction estimated at about 9% among all (recreational) *Cannabis* users (Panlilio et al., 2015), medical controlled applications indicate therapeutic potential and offer the potential for systematic analysis of cannabis use disorder. On the other hand, the inter-entourage effect might be relevant only for very specific combinations of phytocannabinoids and terpenes. According to the literature, inter-entourage effect may be significant despite the fact that terpenes represent a minor component of the total secondary metabolites in *Cannabis* extracts ( ≈ 10%–20%, depending on the extraction method). However, the molecular mechanisms of an inter-entourage effects involving terpenes is still unknown (Namdar et al., 2019).

Clinical studies have not identified psychotropic properties in any cannabinoids other than Δ -(8)9-THC. Consequently, minor cannabinoids show promise based on preclinical studies and initial clinical research (neuropathic pain, neurodegenerative diseases, epilepsy, cancer and skin disorders) (Schonhofen et al., 2018; Walsh et al., 2021). In this context, further research is necessary to bridge the gap in our understanding of minor cannabinoid mechanisms of action and pharmacological effects to fully harness their potential in medical applications.

Cannabinoids are known to interact with a multitude of targets, making them a challenge for *in-vitro* biological evaluations. As an example, studies report Δ -9-THC activity against at least 12 targets (Morales et al., 2017). Previous *in silico* studies explored the currently known cannabinoid targets, their molecular mechanisms, possible synthetic or plant-derived ligands, or the effect of cannabinoids on specific targets and associated conditions (Durdagi et al., 2010; Aviz-Amador et al., 2021; Hourfane et al., 2023). While there are examples of studies focusing on the broader modes of actions of cannabinoids which identify novel targets, they are generally limited to the major cannabinoids (Bian et al., 2019). The present work is therefore focused on the interactions of selected major and minor cannabinoids with a large library of human proteins using inverse molecular docking fingerprints. In inverse molecular docking, a promiscuous ligand is docked against a database of target protein structures, reversing the typical high-throughput virtual screening workflow where a database of ligands is docked into a single protein target. Inverse molecular docking has been successfully applied to obtain mechanistic insights into the adverse side effects of natural compounds (Chen and Ung, 2001; Kores et al., 2021). Moreover, it has proven successful in establishing modes of action and in identifying potential novel targets of natural compounds (Furlan et al., 2018; Lešnik and Bren, 2021; Kores et al., 2022), as well as in drug repurposing (Chen, 2014; Wang et al., 2019; Pinzi et al., 2024; Tanoli et al., 2025). Furthermore, the novel approach of inverse molecular docking fingerprinting has been devised to identify approved drugs with comparable effects on protein targets from the *Coronaviridae* family (Jukič et al., 2021). To the best of our knowledge, the inverse docking studies on a set of cannabinoids against human protein targets has not been conducted yet.

Therefore, the objectives of this study are to further develop the inverse molecular docking fingerprinting method with hierarchical clustering, apply the method on cannabinoids, and suggest novel molecular targets for the studied compounds. We hypothesize that applying inverse docking fingerprinting method will enable us to analyze the similarities and differences in cannabinoid binding patterns and identify structural patterns without prior chemical structure analysis and comparison. We also hypothesize that using this approach, we can suggest potential novel protein targets of cannabinoids and speculate on their mechanisms of action, paving the way for potential novel pharmacologic uses of cannabinoids.

## 2 Methods

### 2.1 Inverse molecular docking

Inverse molecular docking was performed with the ProBiS-Dock (Fine et al., 2020; Konc et al., 2022) software, which employs a hierarchical approach to reconstruct small molecules within protein binding sites, utilizing generalized statistical scoring functions and graph theory. The algorithm begins by fragmenting a ligand into smaller components, which are then docked into protein binding sites using knowledge-based scoring functions. Optimal poses of these fragments are identified and assembled using a fast maximum clique algorithm (Depolli et al., 2013). Throughout this process, iterative dynamics adjusts amino acid conformations to account for protein flexibility and to ensure accurate ligand placement. Finally, a conformation optimization step refines the ligands’ fit in the protein binding cavity. Docking scores, expressed in arbitrary units, approximate relative binding free energies. ProBiS-Dock also incorporates solvent, metal ions, and cofactor interactions, often overlooked by traditional docking methods (Fine et al., 2020; Konc et al., 2022). Inverse molecular docking with the ProBiS-Dock algorithm has been thoroughly validated in previous research through retrospective metrics and redocking studies (Furlan et al., 2018; Kores et al., 2019; Jukič et al., 2021; Lešnik and Bren, 2021; Kores et al., 2021; Kores et al., 2022; Konc et al., 2022). Despite this, the limitations of molecular docking approaches in rank ordering ligand affinity due to simplified, computationally fast statistical scoring functions are well known and also apply in inverse molecular docking. High scoring targets identified in this study should thus be considered appropriately, as an enriched set of potential novel targets for cannabinoids, requiring further more detailed computational or experimental study to establish cannabinoid affinity and a place in cannabinoid modes of action.

One of the main challenges of inverse molecular docking represents obtaining a database of locations of protein binding sites, as limiting the docking space to specific sites streamlines the protocol, reducing both its computational time and complexity (Campbell et al., 2003; Hendlich et al., 2003). We obtained binding-site locations from the human subset of the ProBiS-Dock database (Konc et al., 2021), which automatically prepares non-redundant protein small-molecule binding-sites. The database is constructed from 100% sequence similarity clusters of protein structures from the RCSB PDB (Berman et al., 2000), in which small-molecule binding-sites are identified by binding site comparison using the ProBiS algorithm (Konc and Janežič, 2010; Konc and Janežič, 2017) and by further clustering of results to discern binding-site locations. The ProBiS-Dock database, and the algorithm behind its construction, have been previously successfully applied in inverse molecular docking studies (Kores et al., 2021; Kores er al., 2022; Jukič et al., 2021), and have inspired further tools for the identification of conserved water molecules or metal binding sites in proteins (Jukič et al., 2017; Ravnik et al., 2023). Our study included 55,008 receptor structures and locations of small-ligand binding-sites from the human subset of the database. While the limitations in the composition of the ProBiS-Dock database (which is largely limited by the availability of experimental protein structures) results in certain protein families being over or under-represented (e.g., human GPRs involved in the ECS lack experimental structures), this does not discount the value of an inverse docking experiment on the currently available human structures, the results of which may include some bias towards more highly researched protein families, but should still yield relevant high scoring targets. Protein structures were taken as-is from the ProBiS-Dock database, which retains metallic cofactors but omits organic ones, as well as water molecules. This limits the validity of docking results for enzymes dependent on organic cofactors and presents a future optimization point.

Fourteen cannabinoid compounds are included in this study, representing both major cannabinoids and commonly occurring minor cannabinoids. These include: Δ -9-tetrahydrocannabinol ( Δ -9-THC), tetrahydrocannabivarin (THCV), Δ -8-tetrahydrocannabinol ( Δ -8-THC), cannabinol (CBN), tetrahydrocannabinolic acid (THCA), tetrahydrocannabivarinic acid (THCVA), cannabicyclol (CBL), cannabichromene (CBC), cannabichromenic acid (CBCA), cannabigerol (CBG), cannabigerolic acid (CBGA), cannabidiol (CBD), cannabidivarin (CBDV), cannabidiolic acid (CBDA), and cannabidivarinic acid (CBDVA). We note that both stereoisomers of CBC and CBL-class cannabinoids are produced in *Cannabis* plants (Hanuš et al., 2016), while only a single enantiomer of CBL and CBC is used in the study (Figure 1). The latter were selected for study due to their biological relevance, chemical diversity, and commercial availability. They encompass the most prominent compounds in the cannabis plant, including THC, CBD, and CBG, as well as their precursors (THCA, CBDA, CBGA) and cannabivarin analogs (THCV, CBDV), which offer distinct pharmacological profiles. This series covers a spectrum of psychoactive and non-psychoactive compounds, degradation products, and biosynthetic intermediates. Compounds in this series are among the most researched and provide a foundation for both *in silico* and experimental future research.

**FIGURE 2:**
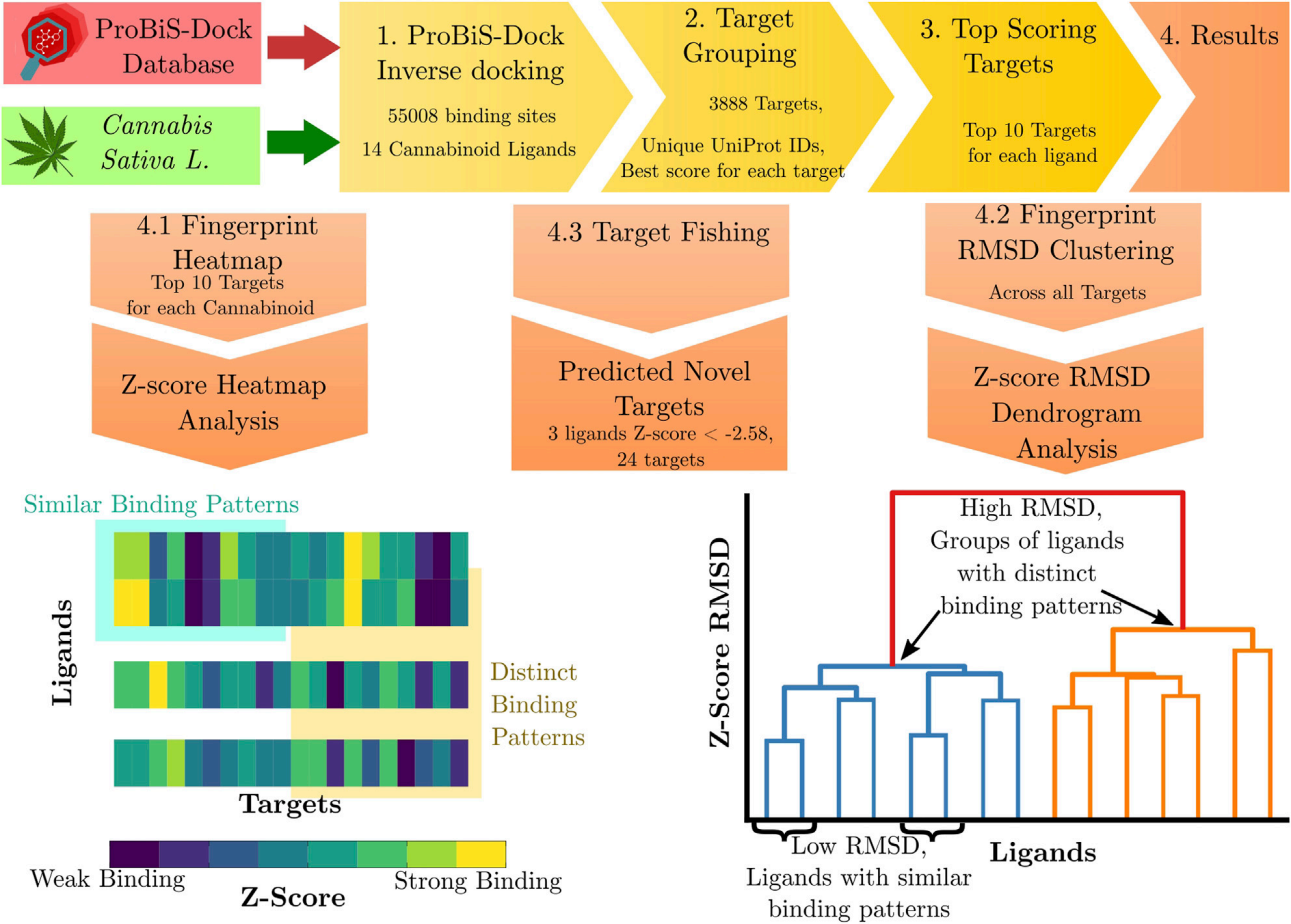
Schematic depiction of the applied methodology. Cannabinoids from *Cannabis Sativa L.* were inversely docked into human protein binding-sites from the ProBiS-Dock database using the ProBiS-Dock. Binding-sites were grouped by UniProt ID, with the best score used for each protein target. Top-scoring proteins were identified as potential novel targets of cannabinoids. Inverse molecular docking fingerprints were applied to compare ligand binding patterns through a heatmap of top-scoring target docking-scores and through clustering based on the RMSD of fingerprints across all protein targets.

Cannabinoid structures were prepared by enumeration of chiral centers, major tautomeric structure selection, removal of structural faults, ionization at the pH of 7.4 and minimization (using OPLS3e force-field) to generate the final 3D conformations used as docking input structures. For this step, LigPrep tool by Schrödinger (Release 2023–4, Schrödinger, LLC, New York, NY, 2025) was employed. Inverse molecular docking results were analyzed using RCSB PDB (Berman et al., 2000) data, mapping PDB IDs and chain identifiers to corresponding UniProt IDs. Since a UniProt ID uniquely represents a specific protein (UniProtConsortium, 2023), we grouped our structures by their UniProt ID and used the best docking score for each ligand if multiple structures shared the same UniProt ID. We note that this is essentially the same methodology as in previous inverse molecular docking studies (Furlan et al., 2018; Kores et al., 2019; Jukič et al., 2021; Lešnik and Bren, 2021; Kores et al., 2021; Kores et al., 2022), except we performed the grouping by protein identity step explicitly before presenting the data. This grouping resulted in 3,888 unique protein targets, with a mean of 13.1 structures per target, and a median of 4. The variation in structure counts per target arises from factors such as structures containing multiple identical chains or minor sequence differences, like length variations or mutations, not captured by 100% sequence identity clustering. Utilizing a larger database of binding sites and consolidating it into unique protein targets may introduce some bias, as targets with more representatives exhibit greater conformational sampling. However, we believe the docking was sufficiently exhaustive to minimize this effect and produce valuable enrichment as we demonstrate with retrospective metric validation of our results. This observation can also enhance the creation of future inverse docking libraries to focus on highly non-redundant sets and de-duplication. For each cannabinoid ligand, we calculated the average and standard deviation of the docking score across all protein targets. For reader clarity, we presented the results in terms of Z-scores, where a negative Z-score represents a stronger interaction, instead of arbitrary docking score units, since the target docking score distribution for each individual ligand can be well described by a normal distribution (see SI). In this manner, the context of the whole dataset scores can be inspected, as different ligands exhibit different average scores, and thus an absolute docking score value is better understood in the context of the whole dataset. Data analysis was performed with the pandas python library (Wes, 2010; pandas development team, 2024). For detailed results in terms of specific structures (PDB ID and chain) as well as docking scores, see the Supplementary SI. Figure 2 depicts a schematic representation of the applied methodology.

We thoroughly analyzed the best-scoring targets in the study, emphasizing those that display consistently favorable Z-scores across several cannabinoids as putative biological targets. We evaluated their potential as promising candidates for further computational or experimental exploration by exploring their therapeutic potential and disease relevance in literature. Furthermore, we have estimated their druggability with the DoGSiteScorer method (Volkamer et al., 2012). DoGSiteScorer is an *in silico* tool used to evaluate binding pockets on protein surfaces by combining geometric and physicochemical analyses. It applies a Difference of Gaussian (DoG) filter and characterizes pockets based on size, shape, depth, and hydrophobicity. Each predicted pocket is then scored (0–1; high values representing high druggability) to help researchers prioritize targets for structure-based drug design.

#### 2.1.1 Inverse molecular docking fingerprints

While the inverse molecular docking methodology has been previously validated and successfully applied for identifying promising protein targets of different ligands (sometimes referred to as target fishing) (Chen and Ung, 2001; Rollinger et al., 2009; Kores et al., 2021; Furlan et al., 2018; Lešnik and Bren, 2021; Kores et al., 2022; Chen, 2014; Wang et al., 2019; Jukič et al., 2021), the authors acknowledge the need for further computational or experimental studies to confirm the relevance of the identified protein targets. Thus, we apply the novel inverse molecular docking fingerprints (Jukič et al., 2021) method as a complementary approach to the traditional inverse molecular docking. As originally introduced, the method applies docking scores on a set of targets to identify similar binding patterns. It examines the binding scores of top scoring targets, and employs them as fingerprints for each compound. The fingerprints are presented as heatmaps where color intensity corresponds to Z-score strength, allowing easy identification of ligand-target binding patterns (see Figure 2 bottom right). For each of the 14 investigated cannabinoids, we selected the top 10 best-scoring targets (combined in a list and removed duplicates) for each cannabinoid to highlight the most biologically relevant interactions, balancing computational manageability and interpretability.

We further manually curated the list and removed non-target proteins that do not represent druggable targets (see SI). Alongside the top scoring targets, we added the known targets of cannabinoids present in the docked database (cannabinoid receptors (CBRs), peroxisome proliferator-activated receptors (PPARs), and transient receptor potential (TRP) channels) to the list (Mackie, 2006). We applied the resulting list of docking Z-scores as the fingerprints of a particular cannabinoid and presented them as a scoring heatmap (Figure 2 bottom right).

This work expands the inverse molecular docking fingerprint methodology by applying agglomerative clustering (Nielsen and Nielsen, 2016) for ligand fingerprint comparison. For this analysis, a ligand fingerprint is derived from docking Z-scores across all targets, instead of focusing solely on the top-ranked ones, as including a larger set improves statistical robustness. The fingerprint for each ligand l , is represented by the array of $N_{\text{t}}$ Z-scores, $Z_{l}$ , where $N_{\text{t}}$ is the total number of protein targets and $Z_{l}\left(i\right)$ represents the Z-score for ligand l with the i -th target. While the order of targets in $Z_{l}$ is arbitrary, consistency across ligands is crucial. To compare the ligand fingerprints, we calculate an *all-against-all* root-mean-square deviation (RMSD) matrix R for each l , k ligand pair, where elements of R represent the RMSD between the Z-scores of different ligands, given by Equation 1.

$$R\left(l,k\right)=RMSD\left(l,k\right)=\sqrt{\frac{1}{N_{\text{t}}}\overset{N_{\text{t}}}{\underset{i=1}{\sum }}{\left(Z_{l}\left(i\right)-Z_{k}\left(i\right)\right)}^{2}}$$ (1)

The RMSD matrix, R , was clustered using agglomerative hierarchical clustering with Ward (minimum variance) linkage (Nielsen and Nielsen, 2016), which was selected because studies often identify it as the best performing on model datasets (Saraçli et al., 2013). The resulting dendrogram reveals clusters of ligands with similar binding patterns (see Figure 2, bottom left). The clustering was carried out using SciPy (Virtanen et al., 2020).

The inverse molecular docking fingerprinting methodology is still in its infancy, and we believe that future studies can expand on the methodology to study different classes of natural and synthetic compounds, and gain valuable insight into the bioactivity trends in the examined chemical space, leading to the repurposing of existing drugs or discovery of novel mechanisms of action.

#### 2.1.2 Method validation

Method validation was carried out using established retrospective metrics, such as receiver-operating characteristics (ROC) curves, enrichment factors (EF10%) (Sheridan et al., 2001), robust initial enhancement (RIE) (Sheridan et al., 2001), Boltzmann-enhanced discrimination of ROC (BEDROC) (Truchon and Bayly, 2007), and total gain (TG) (Empereur-Mot et al., 2015) scores. ROC curves represent a plot of the true (TPF, y -axis) versus the false (FPF, x -axis) positive fractions across threshold values. The area under the ROC curve (AUC) measures the overall predictive performance, with values above 0.5 indicating performance better than random guessing. The RIE, EF10%, TG, and BEDROC scores all quantify the early recognition of experimentally relevant protein targets.

We retrieved data on targets validated through experimental studies of cannabinoid ligands from the ChEMBL database (Zdrazil et al., 2024). We selected representative neutral cannabinoids from different structural classes due to their well-established pharmacological activity and sufficient experimental validation data availability. Therefore, we performed validation for neutral cannabinoids of different classes ( Δ -9-THC, CBD, CBG, CBC). We considered targets with a pChEMBL^1^ score greater than 4 as experimentally relevant (Hofmarcher et al., 2019). The RDKit (Landrum, 2023) python library was used to calculate enrichment curves, ROC AUC, enrichment factors, RIE, and BEDROC (Truchon and Bayly, 2007) (α=20) scores, while TG was calculated via the Screening Explorer web-server (Empereur-Mot et al., 2016), the authors of which introduced the metric. These validation metrics collectively ensure robust predictive performance of our inverse molecular docking approach, increasing confidence in the pharmacological relevance of the identified cannabinoid targets.

## 3 Results and discussion

### 3.1 Inverse molecular docking fingerprints

**FIGURE 3:**
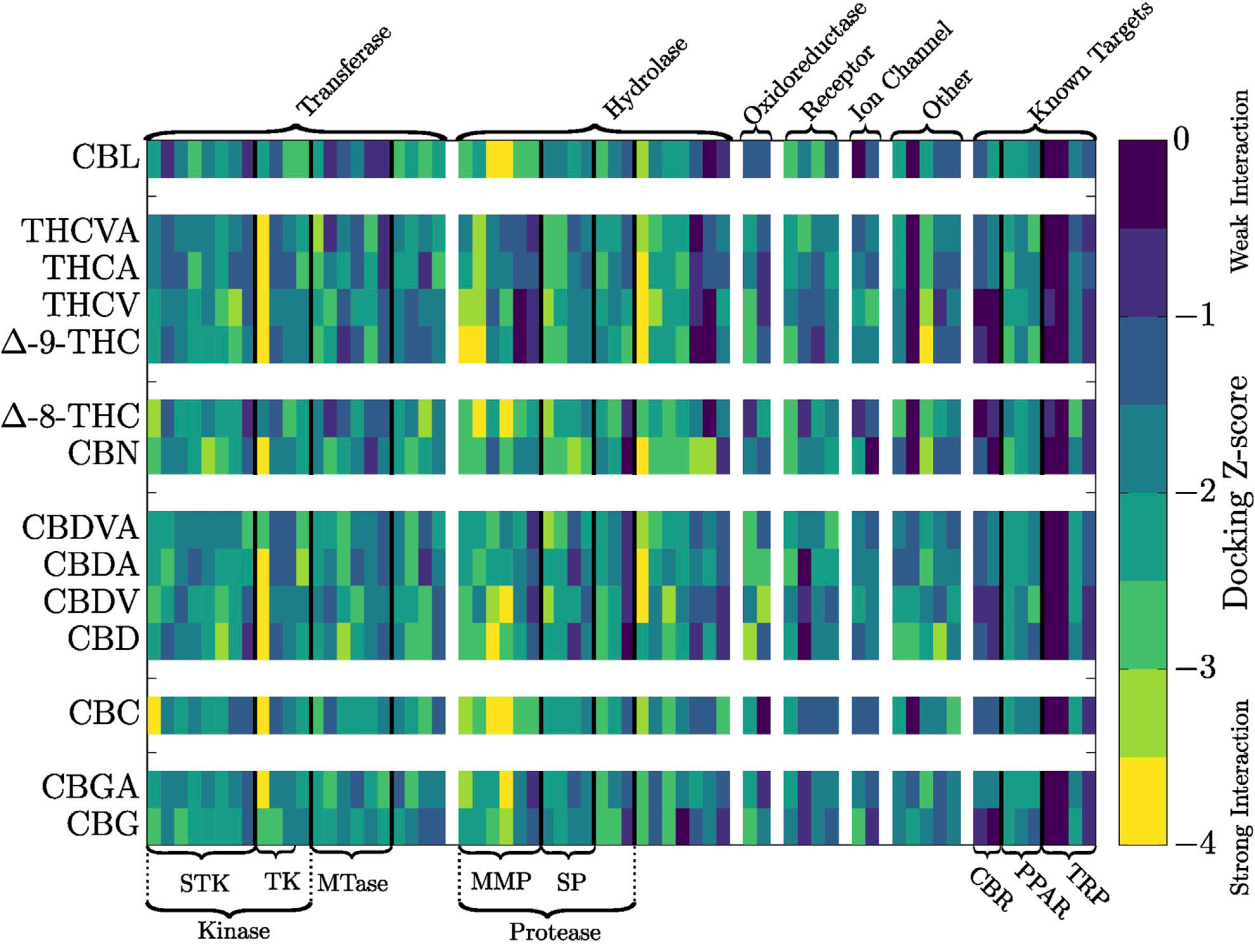
Heatmap of the inverse molecular docking fingerprints of cannabinoids. Yellow regions represent negative Z-scores, indicating predicted favorable interactions. Protein targets are categorized by class, with general classifications above and specific classifications below the heatmap. For more details about the specific targets and numerical values of docking scores, see the Supplementary Material (Supplementary SI; Supplementary Tables S1, S2). Protein classification abbreviations: STK: Serine/threonine-protein kinase, TK: Tyrosine-protein kinases, MTase: Methyltransferase, MMP: Matrix metalloproteinases, SP: Serine proteases, CBR: Cannabinoid receptors, PPAR: Peroxisome proliferator-activated receptors, TRP: Transient receptor potential channels.

To compare the protein targets of different cannabinoids, we employed the inverse molecular docking fingerprint method proposed by Jukič et al. (2021). We combined the top 10 best-scoring protein targets for each of the 14 studied cannabinoids into a single list, removing duplicates. Three non-drug targets were excluded through manual curation (SI). We incorporated nine known cannabinoid targets from the ECS (cannabinoid receptors (Shahbazi et al., 2020), transient receptor potential channels (Muller et al., 2019), and peroxisome proliferator-activated receptors (O’Sullivan, 2016) found in the ProBiS-Dock database, resulting in a curated list of 64 targets. The fingerprint of a cannabinoid is defined as the docking Z-scores against the curated top targets, visualized as a heatmap, Figure 3.

Examining the inverse molecular docking fingerprints heatmap reveals the fingerprints are unique for each compound, hinting at the diversity in target reach across different cannabinoids. Cannabinoids within the same class (Figure 1), which differ only slightly in their chemical structure, exhibit fingerprints with similar binding patterns in certain regions, while other sections of fingerprints remain unique to each individual cannabinoid.

While cannabinoids with variations in alkyl side-chain length (for instance olivetoid vs viridinoid) exhibit highly similar fingerprints, distinct target-specific binding differences are evident. Published research supports this, as shown in a computational docking and molecular dynamics study where Δ -9-THC and THCV displayed similar interactions with CB1 (Jung et al., 2018). However, the main difference was the pentyl side-chain of Δ -9-THC protruding into a secondary pocket, forming an interaction absent in the shorter propyl chain case of THCV, possibly contributing to its reduced CB1 affinity (Jung et al., 2018). Previous experimental research revealed that the THC side-chain length remains crucial for the activity of THC derivatives, with a shorter alkyl chain reducing the affinity of the compound for the cannabinoid receptor (CB1, CB2) (Martin et al., 1999). This shows that subtle structural differences between cannabinoids can result in a significant difference in protein targets, suggesting further study of the mechanisms of action of individual cannabinoid ligands is warranted.

Here we expanded on the inverse molecular docking fingerprint methodology, and provided a quantitative metric of fingerprint comparison, namely, the RMSD between fingerprints, as described above in the Methods section. For greater statistical robustness, RMSD was calculated using docking Z-scores across all docked protein targets, rather than across a subset of top targets. The *“all-against-all”* fingerprint RMSD matrix, R , was analyzed by agglomerative clustering with Ward linkage.

**FIGURE 4:**
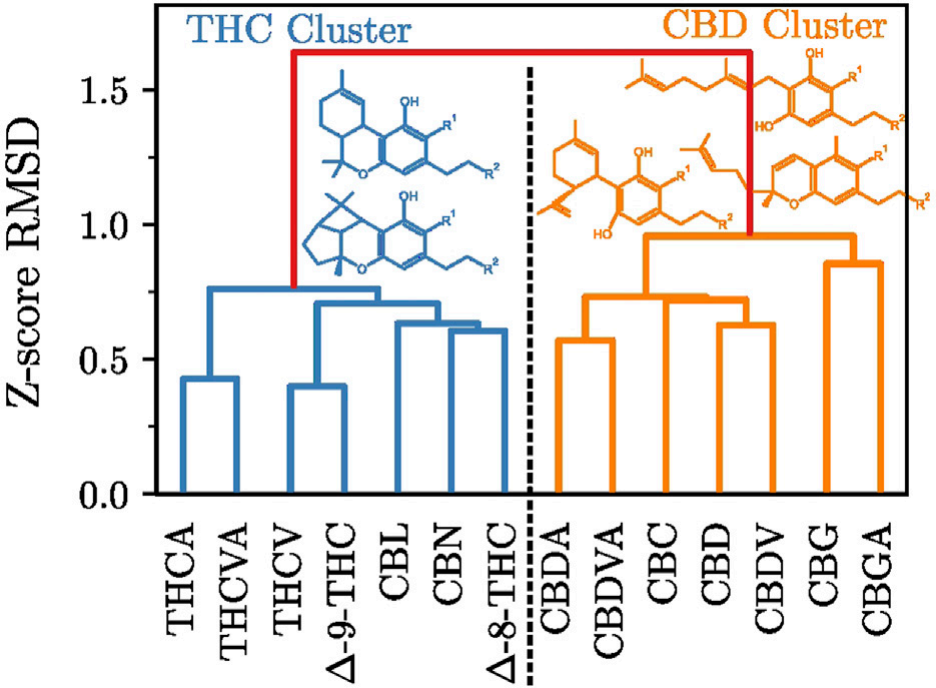
Dendrogram of agglomerative hierarchical clustering with Ward linkage of the all against all ligand-docking Z-score RMSD matrix, R .

The resulting dendrogram (Figure 4) shows two larger clusters of cannabinoid ligands, separated by RMSD>1.5 . The first major cluster contains CBL, Δ -9-THC, Δ -8-THC, and CBN-class cannabinoids, while CBD, CBC, and CBG-class cannabinoids form the second major cluster. We refer to the clusters after their major cannabinoid representative, as the THC cluster and the CBD cluster, respectively.

The THC cluster primarily consists of Δ -9-THC-class compounds and their non-enzymatic conversion products ( Δ -8-THC, CBN). Notably, CBL-class cannabinoids, although derived from CBC-class precursors found in the CBD cluster, display inverse molecular docking fingerprints more similar to THC-class compounds. CBL-class cannabinoids are among the least studied cannabinoid compounds, for example, the ChEMBL database contains a single experimental publication for CBL, in which CBL was tested alongside a number of other cannabinoids (Consroe et al., 1982). More recent studies have flagged CBL as a compound of interest in the treatment of breast cancer (Almeida et al., 2024), as well as an inhibitor of SARS-CoV-2 spike-protein-mediated membrane fusion (Classen et al., 2024). CBL was originally assumed to have a close structural relationship with THC, the compound was therefore designated as THC-III (Korte and Sieper, 1964). Subsequent research, however, revised its structure and traced its origin to the CBC-class compounds (Hanuš et al., 2016). This corroborates our observation that the inverse molecular docking fingerprints of CBL-class cannabinoids are similar to those of THC-class compounds. CBL stands out as particularly promising compound for further study, as the identified binding patterns may lead to some beneficial pharmacological effects similar to THC, while potentially avoiding its adverse side effects (Amin and Ali, 2019) as the little data we have on CBL suggests it lacks narcotic properties (Hanuš et al., 2016). However, the results of this study are preliminary, further research is needed to describe and understand the effects of beneficial or adverse pharmacological effects of CBL.

Within the CBD cluster, CBG-class cannabinoids are further separated from CBC and CBD-class compounds (RMSD≈0.9) , while the differences between CBD and CBC-class compounds are less pronounced (RMSD≈0.75) . We hypothesize that the rigidity of the cannabinoid scaffold plays a role in the observed clustering. The THC cluster contains polycyclic, rigid compounds (THC-class and similar compounds exhibit three condensed rings, while CBL-class compounds exhibit four rings). In contrast, the CBD cluster contains less rigid cannabinoids (CBD and CBC-class cannabinoids feature two rings, while CBG-class compounds feature a single ring). We observed a similar trend in the number of rotatable bonds (excluding the acid group or alkyl side-chain), CBL and THC-class compounds possess four rotatable bonds, CBD-class six, CBC-class seven, and CBG-class ten. These structural differences align well with the observed fingerprint clustering, where more rigid CBL and THC-class compounds form a unified cluster, while more flexible CBD, CBC, and CBG-class form a separate cluster. Moreover, monocyclic CBG-class compounds show a higher RMSD than bicyclic CBD and CBC-class cannabinoids. However, additional future *in-vitro* biological evaluation and *in silico* research is needed to confirm this assertion.

Further analysis of the fingerprint RMSD shows the compounds with the most similar fingerprints differ only in alkyl side-chain length. We have newly observed this trend is also evident in the fingerprint heatmap. Interestingly, the differences in fingerprints between olivetoid and viridinoid cannabinoids are more pronounced for CBD-class compounds (RMSD≈0.6) than for the THC-class cannabinoids (RMSD≈0.4) . This suggests that the alkyl side-chain length exhibits a larger effect on the potential protein targets of CBD-class cannabinoids.

The novel inverse molecular docking fingerprints, paired with clustering, provide a powerful tool for future studies to identify compounds that bind similarly to numerous protein targets and filter out the most promising compounds and avoid “over-experimentation” with all.

Fingerprint clustering can also be applied to smaller sets of curated targets. For example, clustering fingerprints using the curated top targets (Figure 3) still produce a clear THC-CBD cluster division, but the internal cluster relationships vary from the full database fingerprint clustering (see SI, Figure 2).

### 3.2 Potential novel targets of cannabinoids

In addition to examining cannabinoid binding profiles, we analyze and discuss protein targets with highly favorable docking Z-scores as potential novel cannabinoid targets. Table 1 lists all protein targets where at least three cannabinoids exhibit Z-scores below −2.58 (99.5th percentile). We explore the biological and therapeutic relevance of these top-scoring targets and assess their druggability using DoGSiteScorer (Volkamer et al., 2012), reporting the corresponding Drug scores. We report 24 high scoring targets within the Z-score cutoff, all with moderate to high estimated druggability scores (0.72–0.84), and discuss their pharmacological relevance below.

**TABLE 1:** Potential novel cannabinoid targets.

UniProt ID	Name	Connection with disease	Ligands (Z-score <−2.58 )	Drug score^a^
P08631	Hematopoietic cell kinase (HCK)	Cancer	CBC, CBD, CBDA, CBDV, CBDVA, CBG, CBGA, CBLA, CBN, THCA, THCV, THCVA, Δ -9-THC	0.81
P01116	GTPase KRas	Cancer	CBC, CBDA, CBDV, CBDVA, CBG, CBL, CBLA, CBN, THCA, THCV, THCVA, Δ -8-THC, Δ -9-THC	0.80
P11309	Serine/threonine-protein kinase pim-1	Cancer	CBC, CBDV, CBG, CBN, Δ -8-THC	0.80
P61088	Ubiquitin-conjugating enzyme E2 N	Cancer	CBC, CBD, CBDV, CBL, Δ -8-THC	0.72
Q07343	3′,5′-cyclic-AMP phosphodiesterase 4B	Cancer, Neurological diseases, Inflammation	CBLA, CBN, THCA, Δ -9-THC	0.80
P24941	Cyclin-dependent kinase 2	Cancer	CBD, CBN, THCV	0.81
P03372	Estrogen receptor	(Breast) Cancer	CBDA, CBL, CBN, Δ -8-THC, Δ -9-THC	0.81
Q9H9B1	Histone-lysine N-methyltransferase EHMT1	Cancer, Neurological diseases	THCA, THCVA, Δ -9-THC	0.80
Q86X55	Histone-arginine methyltransferase CARM1	Cancer	CBD, CBDA, CBDVA	0.81
Q9NQR1	N-lysine methyltransferase KMT5A	Cancer	CBGA, CBN, THCV	0.78
P14618	Pyruvate kinase PKM, isozyme M2	Cancer	CBDA, CBDVA, CBL, THCA	0.81
O94925	Glutaminase kidney isoform, mitochondrial	Cancer	CBD, CBDV, CBG, CBGA, CBN	0.79
P06737	Glycogen phosphorylase, liver form	Cancer	CBD, CBDA, CBDVA, CBN	0.81
P09467	Fructose-1,6-bisphosphatase 1	Diabetes, Cancer	CBN, THCV, THCVA	0.84
P03956	MMP1 – Interstitial collagenase	Cancer, Arthritis	CBC, CBDA, CBN, THCA, THCV, THCVA, Δ -8-THC, Δ -9-THC	0.81
P45452	MMP13 – Collagenase 3	Cancer, Arthritis	CBC, CBD, CBDV, CBG, CBGA, CBL, THCV, Δ -8-THC	0.84
P08254	MMP3 – Stromelysin-1	Cancer, Arthritis	CBC, CBD, CBDV, CBDVA, CBG, CBL, CBLA	0.75
P14780	MMP9 – Matrix metalloproteinase-9	Cancer, Arthritis	CBC, CBL, Δ -8-THC	0.81
P12830	E-cadherin	Cancer	CBD, CBDV, CBG	0.73
P39900	MMP12 – Macrophage metalloelastase	Inflammatory diseases, Neurological diseases	CBC, CBGA, CBN, THCV, Δ -8-THC, Δ -9-THC	0.74
P27338	Amine oxidase [flavin-containing] B	Parkinson’s disease, Alzheimer’s disease	CBD, CBDA, CBDVA, CBG, THCV, Δ -9-THC	0.81
P56817	Beta-secretase 1	Alzheimer’s disease	CBD, CBDV, CBG, CBL, THCA	0.80
P00742	Coagulation factor X	Blood coagulation	CBDV, CBDVA, CBN, THCA, THCV, THCVA, Δ -8-THC	0.74
P00746	Complement factor D	Complement-driven diseases	CBDVA, CBN, THCA, THCVA, Δ -9-THC	0.80

^a^ Drug score calculated for the primary protein binding site by the DoGSiteScorer Volkamer et al. (2012) web server.

Two targets stand out among the other high scoring targets, exhibiting very favorable interactions with 12 out of the 14 studied cannabinoids, as well as high druggability scores, namely, hematopoietic cell kinase (HCK) and GTPase KRas, both promising anticancer therapeutic targets. HCK represents a non-receptor tyrosine-protein kinase from the SRC family, primarily found in the B-lymphocyte line and myeloid cells. It is involved in numerous processes, including cell differentiation, migration, and proliferation, as well as in regulating cellular homeostasis and the innate immune response via activation of the MAP3K-MAP2K cascade (Chuang et al., 2016). It interacts with the JAK/STAT, RAF/MEK/ERK, PI3K/AKT, CXCL12/CXCR4, among other cellular signaling pathways. Increased HCK activity or dysregulation are linked to the onset or progression of various forms of cancer, including leukemia and solid tumors such as breast, colon, and stomach tumors, indicating HCK represents a promising therapeutic target. Several HCK inhibitory compounds have been identified and studied *in vitro* and *in vivo*, but to date, none of them entered clinical trials (Poh et al., 2015; Luo et al., 2023; Zeng et al., 2024). GPTase KRas serves as a regulator of cellular processes, including cell survival, growth, and differentiation (Yang et al., 2020). The binding of GDP (guanosine diphosphate) or GTP (guanosine triphosphate) serves to switch between the inactive and active forms of KRas, respectively (Zeppa et al., 2024). When activated, KRas triggers downstream signaling cascades, including the RAL, RAF/MEK/ERK, and PI3K/AKT pathways. As a significant oncogenic protein, KRas mutations are present in ≈25% of human cancers, including pancreatic, colorectal, and lung cancers (Kim et al., 2021). KRas was long deemed “undruggable” despite significant research efforts to develop effective anti-KRas therapies (Moore et al., 2020). Recently, studies have identified selective small molecule inhibitors of the *K-RAS G12C* mutant variant, the most prevalent KRas mutation (Uprety and Adjei, 2020). Future studies are needed to determine potential interactions between cannabinoids and mutant KRas variants.

Besides HCK and KRas, other high scoring proteins are also involved in regulating the cell cycle and are connected with tumor growth and, as such, could present important targets in cancer therapy (O’Reilly et al., 2023; Zhong et al., 2023; Laezza et al., 2006). Serine/threonine-protein kinase PIM-1 and cyclin-dependent kinase 2 (CDK2) represent serine/threonine kinases important in cytokine-induced signal transduction (Tadesse et al., 2018; Tursynbay et al., 2016; Zhao et al., 2022). Mutations of estrogen receptor alpha (ESR1) are involved in the majority of breast cancer cases (Grinshpun et al., 2023; Takeda et al., 2013). Furthermore, Lysine methyltransferases EHMT1 (Huang et al., 2010) and KMT5A Lin et al. (2019), as well as arginine methyltranseferase CARM1 (Jin et al., 2023) all exhibit favorable docking scores. They act on histones and can play roles in gene expression and cell cycle progression, and thus represent promising anticancer therapeutic targets. Other highly scoring targets with connections to cancer pathologies include Ubiquitin-conjugating enzyme E2 N (UBE2N; Ubc13), a member of the E2 ubiquitin-conjugating enzyme family. UBE2N is specialized in forming K63-linked polyubiquitin chains, which do not target proteins for degradation. Instead, these chains regulate signaling pathways (Dósa and Csizmadia, 2022; Du et al., 2021; Bui et al., 2021). Despite a lower estimated druggability score (0.72), UBE2N inhibition remains a promising new strategy for anticancer drug development (Schwalen et al., 2025). Phosphodiesterase 4B (PDE4B), another high scoring target, controls cyclic adenosine monophosphate (cAMP) levels. The latter is an important second messenger, elevated levels of which have effects on apoptosis and cell cycle progression in cancer cells (Kim et al., 2019).

Cancer cells require substantial energy to support their proliferation and survival, marking metabolic reprogramming an established hallmark of cancer (Hanahan, 2022). Among the high scoring targets, one finds important metabolic proteins such as glutaminase (GLS), a key enzyme in glutamine metabolism (glutaminolysis), involved in breast cancer (Masisi et al., 2020). Another high scoring target is pyruvate kinase (PKM), which controls the rate limiting step of glycolysis, is expressed in virtually all human cancers (Israelsen and Vander Heiden, 2015). Liver glycogen phosphorylase (PYGL) also features high docking scores with cannabinoids, and is involved in glycogen catabolism and upregulated in glioblastoma (Zois et al., 2022; He et al., 2023). Last but not least, fructose-1,6-bisphosphatase controls gluconeogenesis and acts as a tumor suppressor in breast cancer (Lu et al., 2020).

A prominent group within the top scoring targets are matrix metalloproteinases (MMPs), which include representatives from multiple subgroups: collagenases MMP-1 and MMP-13, stromelysin MMP-3, gelatinase MMP-9, and MMP-12 (Sekhon, 2010). MMPs play a key role in modifying tissue structural integrity and in processing various molecules, including growth factors, proteinases, and their inhibitors, receptors, and adhesion molecules, positioning them as crucial regulators of physiological and pathological processes (Cauwe and Opdenakker, 2010). Studies have shown that changes in MMP levels can exert a large effect on the invasive behavior and formation of tumor metastases (Pytliak et al., 2012). MMPs also play a key role in inflammatory rheumatoid arthritis and osteoarthritis, as their expression is greatly increased in arthritic joints, leading to connective tissue destruction (Burrage et al., 2006). Our results predict multiple cannabinoids exhibit strong interactions with MMPs, CBC and CBL being the most notable, each featuring four MMPs among their ten highest-ranked protein targets (Mustafa et al., 2022; Cabral-Pacheco et al., 2020; Raffetto and Khalil, 2008) (SI). Another high scoring protein target with links to cancer metastasis is E-cadherin, a cell adhesion protein playing an important role in controlling epithelial cell adhesion, movement, and proliferation. It has been shown to play an important role in HP positive gastric cancer and represents a promising therapeutic target, despite only a moderate druggability score (0.73) (Zeng et al., 2015).

The majority of identified top-scoring cannabinoid protein targets are linked to cancer pathologies. To date, cannabinoids have primarily been used as a part of palliative care in cancer patients, and evidence suggests they are effective in alleviating pain and in relieving chemotherapy induced nausea or vomiting (National Academies of Sciences Engineering Medicine, 2017). In conjunction with palliative care, there is a growing body of evidence that cannabinoids exhibit antitumor effects.

The mechanism of action of cannabinoids in cancer is not fully understood. This is especially evident for minor cannabinoids and their actions on targets besides those described for major cannabinoids. Studies on Δ -9-THC suggest a cannabinoid receptor-dependent mechanism of inducing apoptosis and cytotoxicity, while CBD and other cannabinoids act through alternative pathways in the endocannabinoid system. Commonly proposed CBD anticancer mechanisms include the increased production of reactive oxygen species (ROS), leading to cell death via autophagy, preventing the degradation of the endocannabinoid anandamide (AEA), which leads to the inhibition of fatty acid amide hydrolase (FAAH), as well as interactions with other types of receptors (GPR55, TRPV1, TRPV2, TRPM8) (Śledziński et al., 2018; Dariš et al., 2019; Moreno et al., 2019).

While we could not find any current literature evidence for direct interactions between the high scoring targets presented here and cannabinoids, we can identify of them as important contributors in cannabinoid anticancer mechanisms. For example, the PI3K/AKT pathway is an important cell survival mechanism and is regarded among the most common molecular human cancer hallmarks. Research has reported PI3K/AKT inhibition via CB1 receptor interaction with Δ -9-THC (Faiz et al., 2024). Both HCK kinase and GPTase KRAS, which feature very favorable docking scores with most studied cannabinoids, are involved in the PI3K/AKT pathway. CBD and CBN, as well as some synthetic cannabinoids have been shown to induce cell cycle arrest in tumor cells through decreasing levels of key cell cycle components, including CDK2 (O’Reilly et al., 2023; Zhong et al., 2023), for which our results show very favorable docking scores with both CBD and CBN. Δ -9-THC and other synthetic cannabinoids have exhibited regulation of MMP-2 and MMP-9 activity, inhibiting angiogenesis as well as the migratory and invasive capability of cancer cells (Vecera et al., 2020; Pagano et al., 2021). MMP-9 is commonly featured among the top targets in this work, showing very favorable docking scores with Δ -8-THC, CBC, and CBL.

The heterogeneous nature of cancer further complicates this field of study, since different tumor types have been shown to exhibit differing levels of cannabinoid receptors and ECS components (Mangal et al., 2021). The majority of the evidence for anticancer cannabinoid effects comes from studies of the major cannabinoids, while minor cannabinoids remain far less explored (Mangal et al., 2021). The lack of knowledge of minor cannabinoid action, as well as the incomplete understanding of major cannabinoid antitumor activity, provides fertile ground for the exploration of new anticancer cannabinoid targets, which were identified by our inverse molecular docking approach.

Alongside targets involved in cancer pathologies, another group of high-scoring targets could be more broadly related to different neurological diseases. Studies have investigated the involvement of the endocannabinoid system and the effects of cannabinoids on a variety of neurological disorders and find endocannabinoid signalling is altered in most. CBD has recently found success in the treatment of epilepsy (Borowicz-Reutt et al., 2024), while other cannabinoids and ECS components are investigated in a number of neurological disorders including multiple sclerosis, Alzheimer’s, and Parkinson’s diseases (Friedman et al., 2019; Cristino et al., 2020). Among the high scoring targets identified in this study we find MMP-12, which plays a detrimental role in central-nervous system diseases, such as spinal cord injury, stroke, and multiple sclerosis, contributing to their pathogenesis through inflammatory mechanisms (Chelluboina et al., 2018). PDE4B is another high-scoring target, which regulates a range of important functions in the brain, making it a promising therapeutic target for a variety of neurological conditions due to its involvement in modulating neuronal signaling pathway (Blauvelt et al., 2023), interaction with the disrupted-in-schizophrenia 1 (DISC1) protein (Tibbo and Baillie, 2020) and modulation of microglial activity, leading to reduced synaptic loss (Rombaut et al., 2024). Our results show favorable docking scores for EHMT1, studies have found that its inhibition can exert beneficial effects on Alzheimer’s disease (Zheng et al., 2019). Another high scoring target is monoamine oxidase-B (MAOB), which is widely distributed throughout the brain, primarily localized within astrocytes, and is believed to maintain the homeostasis of monoamine neurotransmitters and metabolites in the brain, as well as mediating astrocyte reactivity. MAOB is a key protein in neurodegenerative diseases such as Alzheimer’s and Parkinson’s diseases (Nam et al., 2024; Nam et al., 2022). Beta secretase 1 (BACE1) is the final high scoring target with a connection to neurological disease. It is a membrane-associated aspartic protease involved in the formation of myelin sheaths in peripheral nerves. It cleaves the amyloid precursor protein (APP) and is responsible for the generation of amyloid- β peptides (A β ), which aggregate in the brains of patients with Alzheimer’s disease (Moussa, 2017).

Beyond oncological and neurological targets, an analysis of top targets highlights several proteins with relevance to inflammation. Cannabinoids are known to inhibit inflammatory responses in conditions such as arthritis and multiple sclerosis, mainly by reducing cytokine and chemokine production. They also modulate brain inflammation, although most existing research has centered on major cannabinoids (Klein, 2005; Zurier and Burstein, 2016; McKenna and McDougall, 2020). The most prominent anti-inflammatory group among identified top targets are MMPs, due to their strong link to arthritis. Studies have highlighted the involvement of the endocannabinoid system, especially the CB2 in the pathophysiology of rheumatoid arthritis. CB2 activation may affect arthritis by inhibiting the production of proinflammatory cytokines and MMPs (Gui et al., 2015). MMP-12 also contributes to tissue remodeling in inflammatory respiratory diseases such as chronic obstructive pulmonary diseases (COPD), and represents a promising target for therapies aimed at inflammatory lung diseases (Lagente et al., 2009). A potential anti-inflammatory novel target is HCK kinase, which plays a role in inflammatory processes through NLRP3 inflammasome activation, which promotes the maturation and release of pro-inflammatory cytokines, linking it to the development of inflammatory conditions like type 2 diabetes, atherosclerosis, and Muckle-Wells syndrome (Kong et al., 2020). PDE4B is another potential anti-inflammatory high scoring target. It influences inflammatory response by controlling cAMP levels, which affect production of pro-inflammatory cytokines. It is a critical player in injury-induced neuroinflammation, and its inhibition has been leveraged in approved therapies for inflammatory skin disorders (Tibbo and Baillie, 2020; Blauvelt et al., 2023; Aringer et al., 2024).

Other identified high scoring protein targets include complement factor D, a key player in the alternative complement pathway, one of the three pathways of the complement system, the body’s front-line defense against pathogens. Complement factor D, as the rate-limiting enzyme in the alternative pathway, emerges as a promising target for conditions marked by excessive or dysregulated complement activation (Barratt and Weitz, 2021). Blood coagulation factor X (FX) is another high scoring protein target, which plays a key role in all three pathways of the coagulation cascade, serving as an important driver of thrombin generation and a promising therapeutic target for modulating thrombin production, despite a moderate druggability score (0.74) (Camire, 2021).

The above discussion has demonstrated the pharmacological relevance of the identified high-scoring targets to a variety of conditions: cancer, neurological, and inflammatory disorders. We focus on these particular targets predicting favorable interactions, as well as moderate to high druggability scores. Despite the limitations inherent to the molecular docking methodology, these targets show the potential as novel targets of cannabinoids, and present a possibility for further research to discover new cannabinoid modes of action (MOA). It is important to note that the targets presented here were determined by an arbitrary cutoff, and relevant pharmacological targets may exist beyond it. We therefore provide lists the top targets of individual investigated cannabinoids, as well as comprehensive. csv files of docking scores for all targets in the SI. The Supplementary Material contains a short chapter each individual target in Table 1, the Z-scores of each cannabinoid with the target, and gathered Reactome (Milacic et al., 2024) pathway data. Additionally, lists of top targets of individual investigated cannabinoids, as well as comprehensive. csv files of docking scores for all targets are also included in the SI.

Within the selection of top targets, identifying specific binding patterns between cannabinoid classes and receptors is challenging. While some protein targets exhibit favorable scores with certain cannabinoid types (e.g., PDE4B and EHMT1 with THC cluster cannabinoids, and CARM1 with CBD-class cannabinoids), most receptors do not demonstrate clear cannabinoid–binding patterns (see SI). This further demonstrates the uniqueness of the binding patterns of each cannabinoid. While our inverse molecular docking fingerprints (Figures 3, 4) demonstrate general trends in the target reach of different cannabinoids, the same trends are more difficult to apply to specific receptors. Therefore, further *in vitro* cell and *in silico* studies of individual (minor) cannabinoid action is required for a more thorough understanding of their health promoting effects and applications. While many studies highlight the beneficial synergies between cannabinoids, as well as synergies with the remaining components of *Cannabis* extracts (entourage effect) (Fadda et al., 2004; De Petrocellis et al., 2011; Worth, 2019; André et al., 2024), our study emphasizes the complementary importance of understanding individual cannabinoid targets and MOAs to effectively design tailored cannabinoid-based therapeutic combinations. CBL-class cannabinoids (CBL) specifically stand out as promising candidates requiring further investigation due to the very limited understanding of their properties and effects. Although CBL is rare in *Cannabis*, advancements in (bio)synthetic methods provide opportunities for future studies of CBL and of other less prevalent cannabinoids (Luo et al., 2019; Nguyen et al., 2022; Alfei et al., 2023). We note that the results of this study have focused on binding affinity to potential targets via docking scores, we did not address binding specificity. As cannabinoids are known for their interactions with numerous biological targets, we argue that this analysis can shed some light on possible novel targets which have not been studied to date, alongside the well established targets of the ECS.

### 3.3 Method validation

**FIGURE 5:**
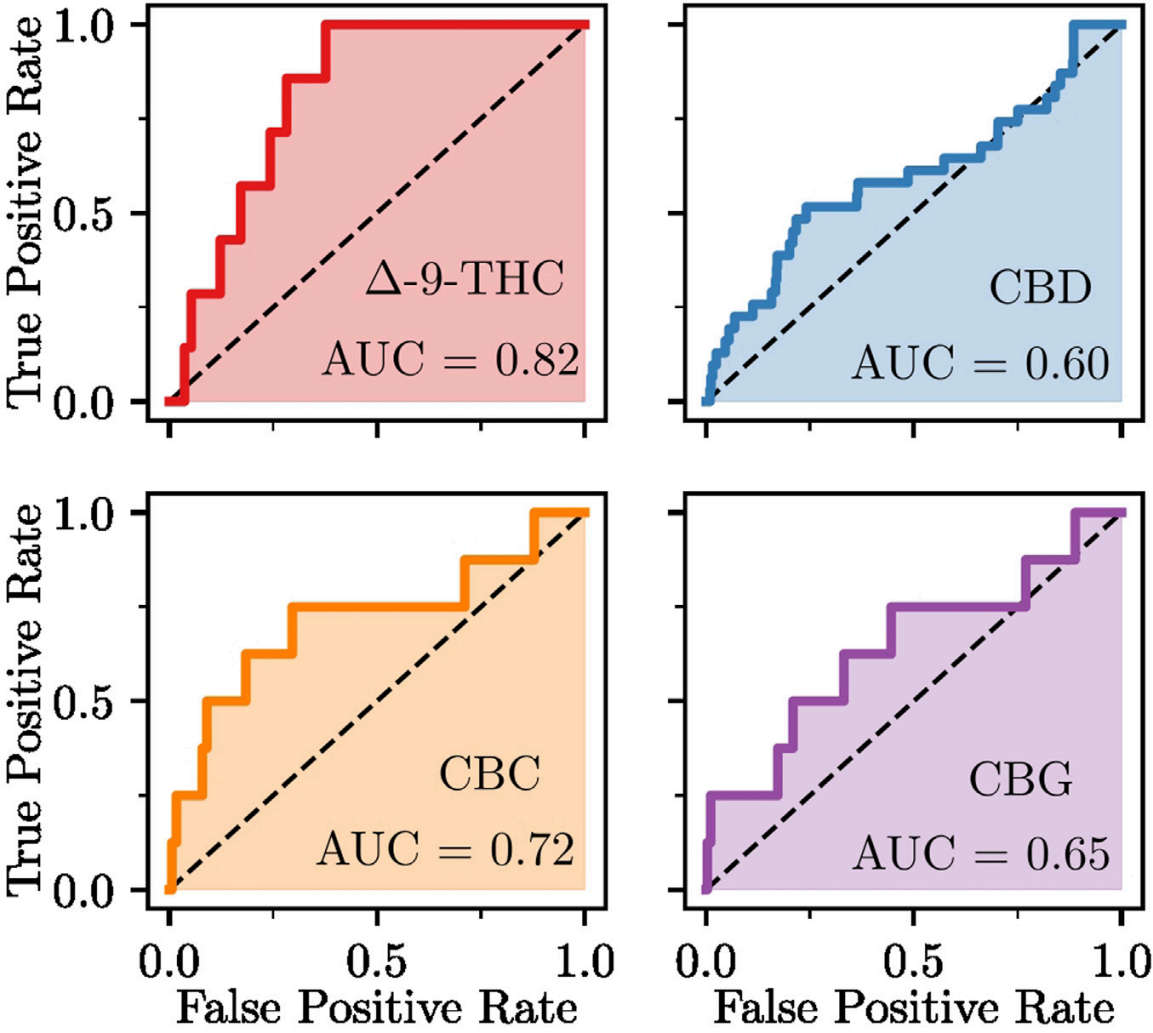
Receiver operating characteristics (ROC) curves for the inverse molecular docking of different cannabinoids.

We evaluated the ability of the applied methodology to distinguish confirmed protein targets of cannabinoids from non-targets. We obtained data on the confirmed protein targets of Δ -9-THC, CBD, CBC, and CBG from the ChEMBL database. The calculated retrospective metrics (Figure 5; Table 2) show that the inverse molecular docking protocol is successful in identifying drug targets. CBD represents the ligand with the least favorable results, with ROC AUC of 0.6 and TG of 0.09. However, other early target detection metrics for CBD, including a RIE of 2.70, EF 10% of 2.26, and BEDROC of 0.15, were more promising. These results, combined with other metrics, validate the protocol’s performance. The retrospective metrics for the remaining cannabinoids were consistently better than for CBD (except for BEDROC of 0.14 for Δ -9-THC).

**TABLE 2:** Retrospective method validation metrics for select cannabinoids.

Ligand	AUC	EF 10%	RIE	BEDROC	TG	$N_{\text{actives}}$
Δ -9-THC	0.82	2.86	2.72	0.14	0.37	7
CBD	0.60	2.26	2.70	0.15	0.09	31
CBC	0.72	5.00	4.93	0.25	0.23	8
CBG	0.65	2.50	4.45	0.23	0.16	8

AUC, Area under the ROC, curve; EF, enrichment factor; RIE, Robust initial enhancement.

BEDROC, Boltzmann-Enhanced Discrimination of ROC; TG, Total gain.

$N_{\text{actives}}$ , Number of identified experimentally relevant targets.

The observed enrichment could be enhanced in future studies via improving the database of docked structures, as well as the docking software itself. Our docked database introduces some bias via unequal representation of target structures, and further de-duplication would likey result in improved enrichment. Furthermore, the presence of mutated structures in the database may also skew the results toward less relevant structures, likely reducing enrichment. Including organic cofactors, conserved water molecules, as well as thorough assignation of protein ionization states all represent possible methodological improvements. Besides database shortcomings, the enrichment is limited by the sampling and scoring function of the docking algorithm, as docking methods often prioritize speed over precision, leading to approximations in both the sampling of conformations and scoring, which may hinder the identification of true positive targets. The latter is especially critical as target bias by specific scoring functions can be introduced. We believe future approaches should benefit by scoring function generalisation and a consensus approach that could address the current shortcomings.

We also examined the docking scores of cannabinoids with the established cannabinoid targets (cannabinoid receptors, peroxisome proliferator-activated receptors, and ion channels, see Figure 3). While the established targets mostly do not appear among the top protein targets for the different cannabinoids, they still predominantly feature favorable docking scores. Based on the described retrospective metrics, we can indeed assert that our methodology identifies relevant high-ranking proteins, even if established targets are not represented among top 10 targets.

## 4 Conclusion

We performed an inverse molecular docking of 14 phytocannabinoid ligands to the exhaustive set of human protein targets from the ProBiS-Dock database and assembled the inverse docking fingerprints. We analyzed the binding patterns of 14 cannabinoids with the inverse molecular docking fingerprints by hierarchical clustering and observed the presence of two large clusters of cannabinoids, indicating similarities in target binding patterns. The first cluster includes THC and related cannabinoids along with CBL-class cannabinoids, while the second cluster comprises CBG, CBC, and CBD-class cannabinoids. Further analysis of cannabinoid fingerprints revealed that while structurally similar cannabinoids share similar binding patterns, unique patterns emerge on a target level. Therefore, while similar cannabinoids may share some affinity for common protein targets, even minor structural differences are enough to change the affinity to specific targets. This suggests that detailed studies of the mechanisms of action of each individual cannabinoid are essential. Our findings suggest that the fingerprints of CBL-class cannabinoids resemble those of THC-class cannabinoids more than their precursors, CBC-class cannabinoids. Due to our lack of understanding of CBL pharmacological effects and the fingerprint similarity with THC, we highlight CBL as a very promising candidate for further experimental studies.

By analyzing the high scoring protein targets of the studied cannabinoids we could speculate on potential novel human targets of minor cannabinoids. We highlight GTPase KRas and hematopoietic cell kinase (HCK), as well as several matrix metalloproteinases (MMPs) as promising candidates for novel cannabinoid targets. Due to the predictive nature of molecular docking results the high scoring targets require further experimental research to confirm their association with cannabinoids.

Minor cannabinoids demonstrate substantial therapeutic potential in computational analyses, especially due to their diverse target-binding patterns and relative underexploration compared to major cannabinoids. However, their therapeutic applications remain to be validated experimentally. Our results provide insight into the similarities in binding patterns of different minor cannabinoids and suggest potential novel protein targets.

The expansion of the inverse molecular docking fingerprinting method with hierarchical clustering represents a powerful tool for future research to analyze and compare the binding patterns of different drug candidates. Moreover, compound structural trends can be identified independently of structural chemoinformatic analysis of input chemical matter, providing insight into examined chemical space and potential pharmacophores. Refining binding site databases by narrowing structure selection criteria, taking into account the presence of mutations, and prioritizing binding sites with experimentally confirmed high-affinity ligands, alongside with further methodological advancements of the inverse molecular docking fingerprinting has the ability to lead to the discovery of novel protein targets, repurposing of existing drugs, and discovery of novel mechanisms of action or potential (adverse) side effects. We firmly believe that this study provides a springboard paving the way for experimental validations *in vitro* and *in vivo*, hopefully leading to novel therapies with cannabinoids.

## Data Availability

The original contributions presented in the study are included in the article/Supplementary Material, further inquiries can be directed to the corresponding author.

## Footnotes

1 pChEMBL score is defined in the ChEMBL database as: $\log_{10}$ (molar IC50, XC50, EC50, AC50, Ki, Kd or Potency).

## Supplementary material

Supplementary Data Sheet 1 Supplementary materials, additional details about the results presented in the article.

Supplementary Data Sheet 2 Raw data, a .csv file for each cannabinoid containing rank ordered protein targets.
