# Supplementary material for: Uncovering the molecular targets of phytocannabinoids: mechanistic insights from inverse molecular docking fingerprint approaches
Source: Front Pharmacol. 2025 Jun 27;16:1611461. doi: 10.3389/fphar.2025.1611461 (PMC12245788; doi:10.3389/fphar.2025.1611461)
Supplement: Supplementary file 1 [file DataSheet1.pdf]

Supplementary Material
Uncovering the Molecular Targets of Phytocannabinoids: Mechanistic Insights from Inverse Docking Fingerprint Approaches

1 SUPPLEMENTARY METHODS

1.1 ProBiS-Dock docking details

In the study we used ProBiS-Dock (Aug. 2022 version) with 20 maximum possible output conformations (max_possible_conf: 20), knowledge based scoring function (probis_level: 0.0) and otherwise default parameters (flex_radius: 8 Å, exhaustiveness: 8...).

1.2 Cannabinoid structure preparation details

Cannabinoid structures were prepared with the LigPrep tool by Schrödinger (Release 2023-4, Schrödinger, LLC, New York, NY, 2025). Cannabinoid structures were ionized at a pH of 7.4 (Epik method, ±1; desalt, check possible tautomers; chiralities were explicitly defined beforehand and retained), resulting in the deprotonation of carboxyl groups in acidic cannabinoids. Initial 3D structures for cannabinoids were generated and minized using the OPLS3e force-field within the LigPrep protocol to produce the docking input structures.

2 FINGERPRINT HEATMAP

Supplementary results for the heatmap fingerprints. Table S1 defines the curated top targets in the fingerprint heatmap (main, Fig. 3).

The list of top targets was manually curated by excluding three targets, based on their biological roles. Somatoliberin (P01286) was excluded due to representing a releasing hormone. Human serum albumin (P02768) was excluded due to being a highly abundant protein in plasma, binding a wide variety of molecules, where binding usually does not reflect therapeutic modulation but rather serves as a pharmacokinetic modulator. Similarly, Hemoglobin subunit beta (P68871) was excluded because it plays an essential physiological role where direct modulation is not immediately warranted.

Table S2 shows numerical docking Z-score values. Docking attempts that failed are assigned a Z-score of $+4$.

Table S1: Curated top targets in fingerprint heatmap (main, Fig. 3)

Index	UniProt ID	Gene	Protein Name	Protein classification
1	P11309	PIM1	Serine/threonine-protein kinase pim-1	Transferase; Kinase; Serine/threonine-protein kinase
2	O15530	PDPK1	3-phosphoinositide-dependent protein kinase 1	Transferase; Kinase; Serine/threonine-protein kinase
3	P15056	BRAF	Serine/threonine-protein kinase B-raf	Transferase; Kinase; Serine/threonine-protein kinase
4	P68400	CSNK2A1	Casein kinase II subunit alpha	Transferase; Kinase; Serine/threonine-protein kinase
5	Q16539	MAPK14	Mitogen-activated protein kinase 14	Transferase; Kinase; Serine/threonine-protein kinase
6	P24941	CDK2	Cyclin-dependent kinase 2	Transferase; Kinase; Serine/threonine-protein kinase
7	P23443	RPS6KB1	Ribosomal protein S6 kinase beta-1	Transferase; Kinase; Serine/threonine-protein kinase
8	O94804	STK10	Serine/threonine-protein kinase 10	Transferase; Kinase; Serine/threonine-protein kinase
9	P08631	HCK	Tyrosine-protein kinase HCK	Transferase; Kinase; Tyrosine-protein kinase
10	P43405	SYK	Tyrosine-protein kinase SYK	Transferase; Kinase; Tyrosine-protein kinase
11	Q06187	BTK	Tyrosine-protein kinase BTK	Transferase; Kinase; Tyrosine-protein kinase
12	P14618	PKM	Pyruvate kinase PKM	Transferase; Kinase
13	Q9H9B1	EHMT1	Histone-lysine N-methyltransferase EHMT1	Transferase; Methyltransferase
14	Q9NQR1	KMT5A	N-lysine methyltransferase KMT5A	Transferase; Methyltransferase
15	Q86X55	CARM1	Histone-arginine methyltransferase CARM1	Transferase; Methyltransferase
16	Q9NRG4	SMYD2	N-lysine methyltransferase SMYD2	Transferase; Methyltransferase
17	O14744	PRMT5	Protein arginine N-methyltransferase 5	Transferase; Methyltransferase
18	O75530	EED	Polycomb protein EED	Transferase; Methyltransferase
19	P09874	PARP1	Poly [ADP-ribose] polymerase 1	Transferase; Nucleotidyltransferase
20	P06737	PYGL	Glycogen phosphorylase, liver form	Transferase; Glycosyltransferase

Continued on next page

Table S1: Curated top targets in fingerprint heatmap (main, Fig. 3)

Index	UniProt ID	Gene	Protein Name	Protein classification
21	P61088	UBE2N	Ubiquitin-conjugating enzyme E2 N	Transferase; Acyltransferase
22	Q8IXJ6	SIRT2	NAD-dependent protein deacetylase sirtuin-2	Transferase
23	P39900	MMP12	MMP-12, Macrophage metalloelastase	Hydrolase; Protease; Metalloprotease
24	P03956	MMP1	MMP-1, Interstitial collagenase	Hydrolase; Protease; Metalloprotease
25	P08254	MMP3	MMP-3, Stromelysin-1	Hydrolase; Protease; Metalloprotease
26	P45452	MMP13	MMP-13, Collagenase 3	Hydrolase; Protease; Metalloprotease
27	P14780	MMP9	MMP-9, Matrix metalloproteinase-9	Hydrolase; Protease; Metalloprotease
28	P22894	MMP8	MMP-8, Neutrophil collagenase	Hydrolase; Protease; Metalloprotease
29	P00742	F10	Coagulation factor X	Hydrolase; Protease; Serine protease
30	P00746	CFD	Complement factor D	Hydrolase; Protease; Serine protease
31	P00747	PLG	Plasminogen	Hydrolase; Protease; Serine protease
32	P08709	F7	Coagulation factor VII	Hydrolase; Protease; Serine protease
33	P56817	BACE1	Beta-secretase 1	Hydrolase; Protease; Aspartyl protease
34	P00797	REN	Renin	Hydrolase; Protease; Aspartyl protease
35	Q6P179	ERAP2	Endoplasmic reticulum aminopeptidase 2	Hydrolase; Protease; Aminopeptidase
36	P01116	KRAS	GTPase KRas	Hydrolase
37	P09467	FBP1	Fructose-1,6-bisphosphatase 1	Hydrolase
38	O94925	GLS	Glutaminase kidney isoform, mitochondrial	Hydrolase
39	Q07343	PDE4B	3',5'-cyclic-AMP phosphodiesterase 4B	Hydrolase
40	P62333	PSMC6	26S proteasome regulatory subunit 10B	Hydrolase
41	O75608	LYPLA1	Acyl-protein thioesterase 1	Hydrolase
42	O14732	IMPA2	Inositol monophosphatase 2	Hydrolase
43	P27338	MAOB	Amine oxidase [flavin-containing] B	Oxidoreductase
44	P48735	IDH2	Isocitrate dehydrogenase [NADP], mitochondrial	Oxidoreductase
45	P03372	ESR1	Estrogen receptor	Receptor

Continued on next page

Table S1: Curated top targets in fingerprint heatmap (main, Fig. 3)

Index	UniProt ID	Gene	Protein Name	Protein classification
46	P28702	RXRB	Retinoic acid receptor RXR-beta	Receptor
47	Q92731	ESR2	Estrogen receptor beta	Receptor
48	P55055	NR1H2	Oxysterols receptor LXR-beta	Receptor
49	Q9Y698	CACNG2	Voltage-dependent calcium channel gamma-2 subunit	Ion channel
50	P17787	CHRNB2	Neuronal acetylcholine receptor subunit beta-2	Ion channel
51	P62937	PPIA	Peptidyl-prolyl cis-trans isomerase A	Other; Isomerase; Rotamase
52	Q96GA7	SDSL	Serine dehydratase-like	Other; Lyase
53	P12830	CDH1	Cadherin-1	Other; Cell adhesion
54	Q16658	FSCN1	Fascin	Other; Actin-binding
55	P61769	B2M	Beta-2-microglobulin	Other; Immune system
56	P21554	CNR1	Cannabinoid receptor 1	Known Target; Receptor; G-protein coupled receptor
57	P34972	CNR2	Cannabinoid receptor 2	Known Target; Receptor; G-protein coupled receptor
58	P37231	PPARG	Peroxisome proliferator-activated receptor gamma	Known Target; Receptor
59	Q03181	PPARD	Peroxisome proliferator-activated receptor delta	Known Target; Receptor
60	Q07869	PPARA	Peroxisome proliferator activated receptor alpha	Known Target; Receptor
61	Q9HBA0	TRPV4	Transient receptor potential cation channel subfamily V member 4	Known Target; Ion channel
62	Q8NER1	TRPV1	Transient receptor potential cation channel subfamily V member 1	Known Target; Ion channel
63	O75762	TRPA1	Transient receptor potential cation channel subfamily A member 1	Known Target; Ion channel
64	Q8NET8	TRPV3	Transient receptor potential cation channel subfamily V member 3	Known Target; Ion channel

Table S2: Z-score values in fingerprint heatmap (main, Fig. 3).

Gene	CBL	THC-VA	THCA	THCV	Δ-9-THC	Δ-8-THC	CBN	CBD-VA	CBDA	CBDV	CBD	CBC	CBGA	CBG
PIM1	-2.05	-1.99	-1.92	-2.21	-2.19	-3.12	-2.72	-2.06	-2.04	-2.65	-2.46	-3.64	-2.19	-2.78
PDPK1	-0.84	-1.39	-1.47	-1.57	-1.33	-1.26	-1.61	-2.40	-2.80	-2.29	-1.32	-1.94	-1.81	-1.84
BRAF	-1.80	-1.82	-1.49	-1.51	-1.67	-2.27	-1.93	-1.68	-1.79	-1.43	-1.82	-2.10	-1.68	-2.75
CSNK2A1	-2.52	-1.56	-2.82	-2.21	-2.31	-2.16	-2.24	-1.70	-1.30	-2.42	-1.95	-1.95	-2.05	-2.19
MAPK14	-1.88	-1.55	-1.99	-1.81	-2.40	-1.78	-3.05	-1.84	-1.80	-2.03	-2.07	-2.07	-1.90	-2.06
CDK2	-2.31	-2.32	-2.36	-2.74	-2.42	-2.40	-2.79	-1.99	-2.39	-2.56	-3.05	-2.48	-2.40	-2.40
RPS6KB1	-1.58	-1.67	-1.48	-3.29	-2.59	-2.19	-2.49	-1.68	-2.23	-2.06	-2.34	-1.45	-1.87	-2.11
STK10	-0.87	-1.19	-1.24	-1.43	-1.78	-0.91	-1.04	-2.69	-2.38	-1.17	-0.82	-1.20	-1.15	-1.46
HCK	-2.43	-4.87	-5.56	-6.12	-6.13	-1.91	-4.20	-2.77	-3.62	-4.13	-4.15	-4.95	-4.37	-2.65
SYK	-1.11	-1.17	-1.38	-1.56	-1.49	-1.10	-1.92	-1.31	-1.40	-1.51	-1.40	-1.26	-1.79	-2.90
BTK	-2.90	-1.61	-1.29	-1.58	-1.74	-2.69	-1.86	-1.48	-1.42	-1.72	-1.98	-1.96	-1.81	-1.65
PKM	-2.70	-2.18	-2.69	-1.68	-1.76	-2.30	-2.37	-2.73	-3.50	-1.79	-2.11	-2.03	-2.03	-1.83
EHMT1	-2.36	-3.11	-2.89	-2.44	-2.78	-1.57	-1.90	-2.25	-2.24	-1.97	-2.41	-2.56	-2.49	-2.36
KMT5A	-0.74	-0.87	-1.62	-2.76	-2.34	-0.86	-2.79	-2.13	-1.98	-1.38	-1.90	-1.34	-2.78	-2.23
CARM1	-1.31	-1.70	-2.36	-1.02	-0.93	-1.15	-2.07	-2.71	-2.66	-2.42	-3.08	-2.47	-1.96	-1.75
SMYD2	-1.64	-1.09	-1.08	-2.11	-1.33	-2.37	-1.60	-1.79	-1.43	-2.97	-2.19	-2.28	-1.06	-2.17
PRMT5	-0.81	-2.67	-2.23	-2.20	-2.64	-1.24	-0.74	-1.77	-1.76	-1.82	-1.88	-2.15	-2.12	-1.53
EED	-0.55	-0.95	-0.79	-1.45	-1.17	-1.22	-1.61	-1.17	-1.17	-1.79	-1.29	-1.57	-2.52	-2.07
PARP1	-2.72	-1.97	-2.21	-1.70	-1.97	-2.08	-2.19	-1.63	-1.52	-2.02	-1.76	-1.62	-1.29	-2.19
PYGL	-2.39	-2.32	-2.26	-1.27	-1.25	-1.69	-2.67	-2.90	-2.85	-2.06	-2.86	-2.13	-2.51	-1.75
UBE2N	-2.83	-1.95	-0.77	-1.68	-1.10	-3.15	-2.04	-1.16	-0.90	-2.88	-2.96	-2.92	-1.53	-1.40
SIRT2	-2.08	-2.41	-2.87	-1.56	-1.81	-1.89	-1.77	-2.33	-1.71	-1.32	-1.42	-1.45	-1.99	-1.17
MMP12	-2.53	-1.65	-2.02	-3.26	-4.07	-2.91	-2.80	-2.19	-2.21	-2.53	-2.51	-3.16	-3.03	-2.13
MMP1	-2.37	-3.00	-3.03	-3.15	-4.54	-3.79	-2.63	-2.45	-2.81	-1.88	-2.51	-2.99	-2.22	-2.32
MMP3	-3.66	-1.60	-1.72	-1.20	-1.53	-2.40	-1.38	-2.70	-2.23	-3.39	-3.62	-3.92	-2.46	-2.81
MMP13	-3.60	-1.34	-1.82	-2.73	-2.49	-5.31	-2.56	-1.85	-2.25	-3.53	-2.85	-4.36	-3.57	-3.29
MMP9	-2.92	-1.03	-1.10	-0.38	-0.27	-2.70	-1.94	-2.03	-1.85	-1.69	-2.47	-2.68	-1.99	-1.83
MMP8	-2.98	-0.82	-1.11	-0.57	-0.79	-2.12	-1.52	-0.72	-1.32	-0.85	-1.25	-2.59	-0.87	-1.47

Continued on next page

Table S2: Z-score values in fingerprint heatmap (main, Fig. 3).

Gene	CBL	THC-VA	THCA	THCV	Δ-9-THC	Δ-8-THC	CBN	CBD-VA	CBDA	CBDV	CBD	CBC	CBGA	CBG
F10	-1.99	-2.87	-2.97	-3.17	-2.54	-3.04	-2.61	-3.16	-2.47	-3.22	-2.38	-2.11	-2.10	-1.86
CFD	-1.76	-2.76	-2.93	-2.26	-2.90	-2.44	-2.73	-2.61	-2.30	-2.41	-2.38	-2.43	-2.32	-2.03
PLG	-2.29	-1.41	-1.52	-1.53	-1.56	-2.31	-3.04	-1.40	-0.85	-2.27	-0.61	-2.07	-1.29	-1.91
F7	-1.95	-1.73	-1.35	-1.82	-1.85	-1.92	-2.92	-2.40	-1.94	-1.41	-1.98	-1.75	-1.97	-1.96
BACE1	-2.89	-2.05	-2.61	-2.03	-1.83	-2.34	-1.96	-2.27	-2.29	-2.94	-2.74	-2.57	-2.52	-2.83
REN	-1.84	-2.06	-1.71	-1.98	-2.24	-2.57	-2.07	-1.58	-1.76	-2.25	-2.45	-2.30	-1.77	-2.91
ERAP2	-1.25	-1.25	-1.12	-2.27	-2.77	-0.67	-0.33	-0.68	-0.85	-0.72	-0.40	-1.33	-1.36	-0.66
KRAS	-3.00	-3.49	-3.62	-4.57	-3.75	-2.81	-3.66	-3.10	-3.98	-3.83	-2.40	-3.43	-2.52	-2.76
FBP1	-1.72	-2.72	-2.35	-3.37	-2.40	-2.34	-2.80	-2.52	-2.33	-1.81	-1.73	-1.99	-1.22	-1.25
GLS	-2.21	-2.25	-2.33	-2.10	-2.24	-2.52	-2.79	-2.24	-1.93	-3.07	-2.82	-2.56	-2.88	-2.72
PDE4B	-2.26	-2.03	-2.92	-2.35	-2.61	-2.44	-2.77	-2.15	-2.21	-1.74	-2.47	-2.00	-1.66	0.13
PSMC6	-1.15	-0.45	-0.85	-0.57	-0.47	-1.98	-3.08	-1.38	-1.96	-1.30	-0.89	-1.28	-2.37	-1.34
LYPLA1	0.34	-1.10	-1.15	0.32	0.16	0.20	-3.16	-1.61	-2.29	-1.44	-2.17	-1.12	-1.58	-1.61
IMPA2	-0.97	-1.61	-1.31	-2.10	-1.84	-1.79	-0.99	-1.13	-1.49	-0.98	-0.77	-2.77	-1.15	-1.00
MAOB	-1.07	-2.37	-1.77	-2.70	-2.88	-0.70	-1.57	-2.95	-2.67	-1.74	-3.12	-2.46	-2.44	-2.58
IDH2	-1.37	-1.29	-0.83	-0.58	-1.36	-2.07	-1.42	-1.06	-2.83	-3.24	-1.32	-0.48	-0.82	-1.95
ESR1	-2.69	-2.16	-2.12	-1.87	-2.91	-2.58	-2.62	-2.23	-2.73	-1.90	-2.19	-2.20	-2.27	-1.92
RXRB	-1.61	-2.61	-1.86	-1.33	-1.25	-0.93	-1.83	-1.80	0.07	-0.78	-0.33	-1.38	-0.51	-0.99
ESR2	-2.99	-1.87	-1.32	-1.41	-0.95	-2.19	-1.99	-1.93	-2.12	-1.90	-1.58	-1.12	-1.96	-1.66
NR1H2	-1.43	-1.99	-2.06	-1.73	-1.90	-1.76	-2.22	-2.64	-2.14	-1.40	-1.80	-1.50	-2.00	-1.46
CACNG2	-0.49	-1.09	-1.57	-2.44	-1.89	-0.78	-2.17	-1.68	-1.93	-2.24	-1.47	-1.31	-1.78	-2.78
CHRNB2	-1.18	-1.52	-1.13	-2.95	-1.77	-1.22	-0.32	-1.06	-1.91	-1.80	-1.51	-1.16	-1.40	-0.97
PPIA	-2.00	-2.13	-2.37	-1.66	-2.00	-2.81	-1.47	-1.76	-1.29	-2.31	-2.93	-2.31	-1.51	-1.92
SDSL	4.00	4.00	4.00	4.00	4.00	4.00	4.00	-1.13	-1.43	-2.10	-2.94	-0.28	-1.44	-2.00
CDH1	-2.25	-2.87	-3.00	-3.24	-3.53	-2.61	-3.06	-2.46	-2.94	-1.90	-2.08	-1.87	-2.52	-1.74
FSCN1	-1.48	-1.85	-1.05	-0.92	-1.39	-2.05	-1.32	-1.94	-1.93	-1.39	-3.26	-1.54	-1.17	-1.43
B2M	-1.38	-1.70	-1.41	-1.60	-1.22	-1.86	-1.28	-1.31	-1.57	-2.12	-1.98	-2.82	-1.92	-1.70
CNR1	-1.00	-1.18	-1.23	-0.45	-0.83	-0.15	-1.17	-1.07	-1.35	-0.91	-1.10	-1.24	-1.60	-0.88

Continued on next page

Table S2: Z-score values in fingerprint heatmap (main, Fig. 3).

Gene	CBL	THC-VA	THCA	THCV	Δ-9-THC	Δ-8-THC	CBN	CBD-VA	CBDA	CBDV	CBD	CBC	CBGA	CBG
CNR2	-2.42	-1.53	-1.57	-0.33	-0.44	-0.65	-0.42	-1.65	-1.85	-0.82	-0.85	-1.32	-1.92	-0.36
PPARG	-2.10	-2.39	-2.52	-2.23	-2.55	-1.93	-2.66	-2.16	-2.25	-2.29	-2.06	-2.39	-2.34	-2.03
PPARD	-2.31	-1.93	-1.72	-2.30	-1.88	-2.42	-2.08	-2.30	-2.13	-1.60	-1.74	-1.55	-2.18	-1.53
PPARA	-1.85	-2.56	-1.66	-1.54	-2.55	-1.64	-1.90	-1.71	-1.63	-2.58	-1.33	-1.51	-2.00	-1.26
TRPV4	0.04	-0.23	-0.11	-0.94	-0.40	-0.55	-0.43	0.39	0.38	-0.14	0.38	-0.43	0.29	-0.04
TRPV1	0.78	0.67	1.27	1.02	1.23	1.23	0.79	0.99	1.00	0.74	0.81	0.88	0.81	1.11
TRPA1	-1.85	-1.40	-1.51	-1.55	-1.94	-2.52	-1.82	-2.31	-2.06	-2.32	-1.82	-2.02	-1.57	-2.48
TRPV3	-1.05	-0.99	-1.34	-0.60	-0.58	-0.90	-0.91	-1.35	-1.31	-0.65	-0.95	-0.80	-0.98	-0.72

3 FINGERPRINT CLUSTERING

Supplementary results for fingerprint clustering.

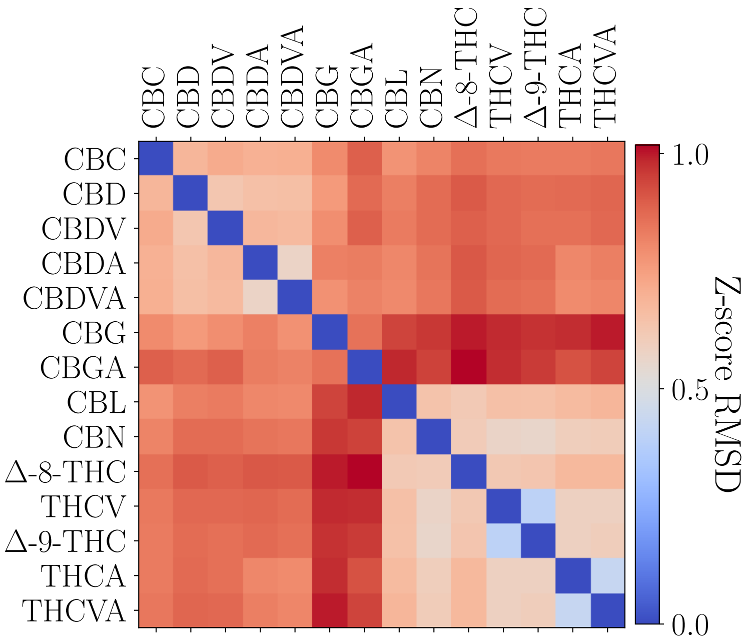

Figure S1. Heatmap plot of the all against all ligand docking Z-score RMSD matrix, $\mathcal{R}$. Results for fingerprints with all targets.

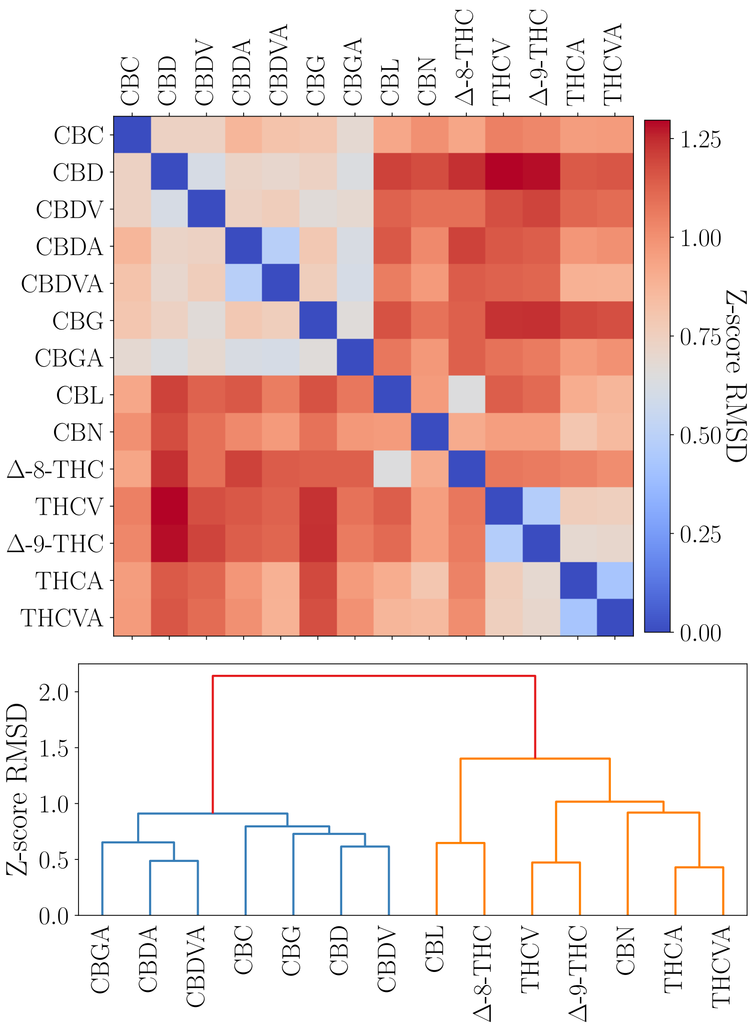

Figure S2. Above: Heatmap plot of the all against all ligand docking Z-score RMSD matrix, $\mathcal{R}$. Below: Dendrogram of agglomerative hierarchical clustering with Ward linkage of $\mathcal{R}$. Results for fingerprints defined by the curated set of top targets (main Fig. 3, Tables S1,S2)

4 PROTEIN CATEGORIES

We have classified proteins in our database according to the UniProt UniProtConsortium (2023) Molecular Function keywords. Figure S3 shows the number of proteins in our database belonging to each (topmost) UniProt molecular function keyword (with a cutoff of 50 targets in our docked database). We observe that our database features a large number of hydrolase and transferase proteins (750+), while receptors, oxydoreductase and DNA-binding proteins are the next most common (350+).

We analyzed the average docking Z-scores for THC and CBD cluster (see main text, Figure 4) cannabinoids for targets of different categories, featuring certain Molecular Function keywords in the UniProt database. Results show little difference between CBD and THC cluster average Z-scores, especially given the large standard deviations of the results. We conclude that while we can use our inverse molecular docking fingerprint clustering method to show differences in target binding patterns, the differences are not sufficiently pronounced to draw conclusions at the level of whole protein families. Overall, cannabinoids represent family of relatively similar ligands. We expect that, including compounds from other structural families in the fingerprint clustering would likely reveal greater differences between those ligands and

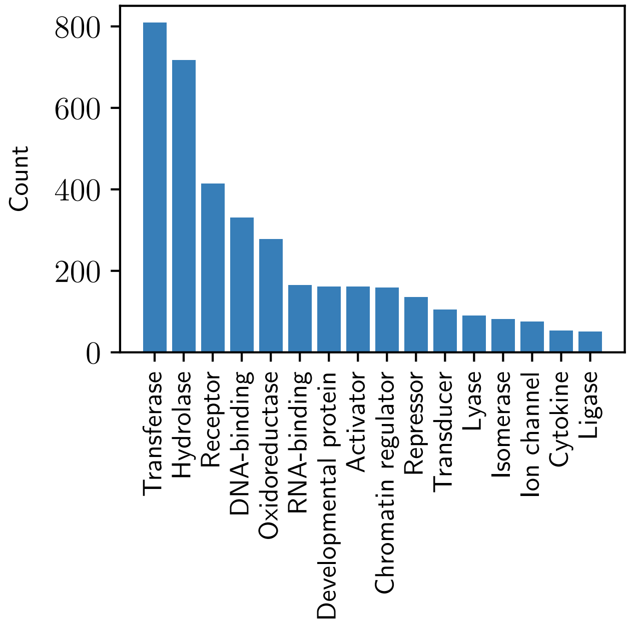

Figure S3. Number of protein targets in our docked database that exhibit certain Molecular Function keywords

cannabinoids than among the cannabinoids themselves. While the differences between the average Z-scores between THC and CBD cluster cannabinoids are too minor to be significant, we can observe patterns for cannabinoids in general. The result show that cannabinoids exhibit favorable interactions (Z-score< -0.5) with kinases (both Serine/threonine and Tyrosine), glycosyltransferases and serine proteases.

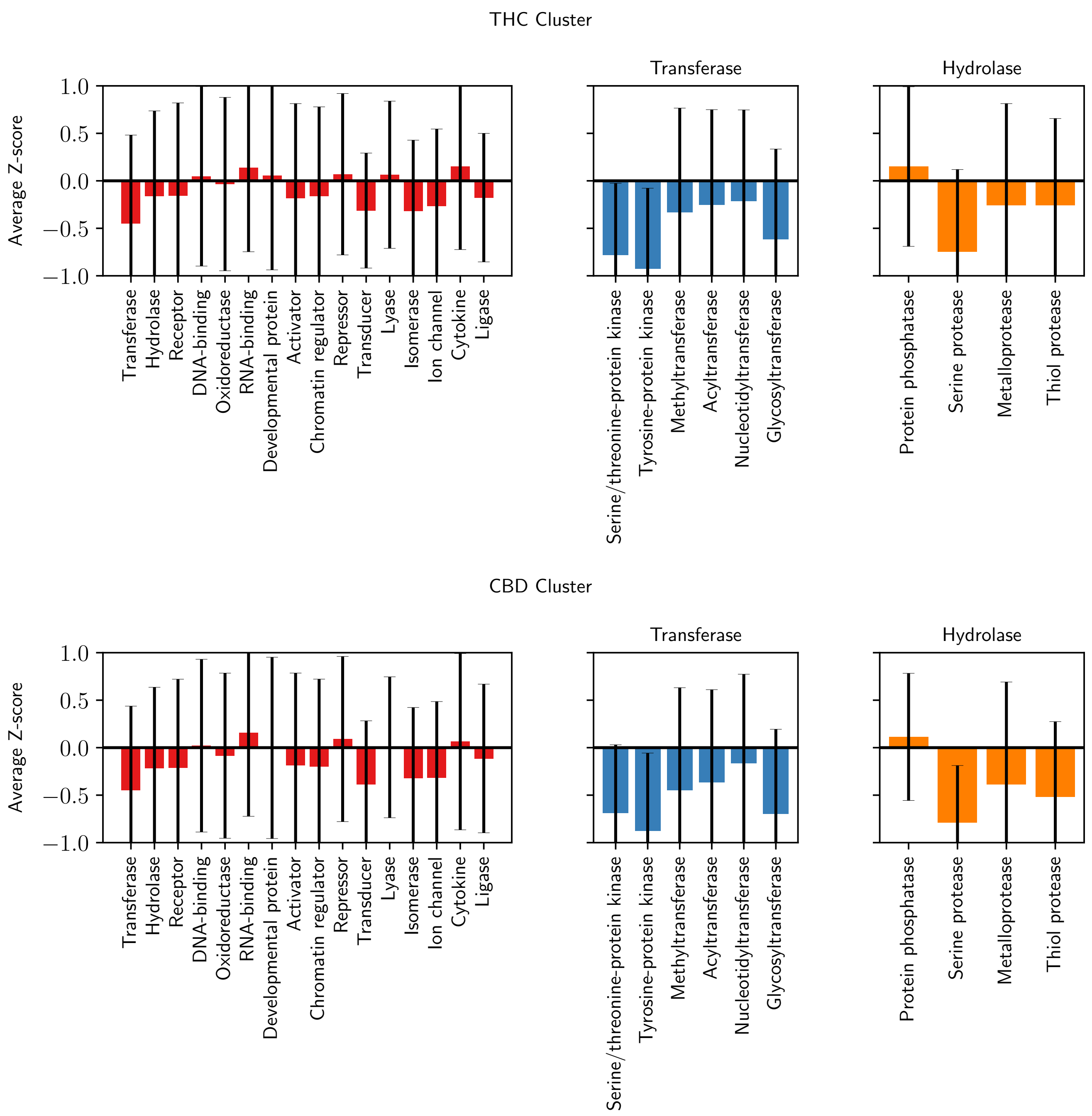

Figure S4. Average docking Z-score for THC (above) and CBD (below) cluster cannabinoids across all targets featuring certain Molecular Function keywords. Broad categories (topmost keywords) are plotted in red, subgroups of the Transferase and Hydrolase proteins are plotted in blue and orange, respectively. Standard deviations of Z-scores are represented with black lines.

5 POTENTIAL NOVEL TARGETS OF CANNABINOIDS

This section presents an extended version of the main text Table 1. The section features a short description of each protein discussed in the main text and its function, as well as associated references. Furthermore, this section features a bar plot of the docking Z-scores for all investigated cannabinoids for each of the targets,

as well a table featuring biological pathway data from the Reactome pathway knowledgebase Milacic et al. (2024).

5.1 Hematopoietic cell kinase (HCK)

Protein UniProt ID: P08631

Protein PDB ID-Chain: 2C0T-A

Non-receptor tyrosine-protein kinase, important in regulation of innate immune responses and cellular homeostasis, participating in cell differentiation, migration, and proliferation via the secretion of growth factors and cytokines Poh et al. (2015); Luo et al. (2023); Zeng et al. (2024).

Connection with disease: Cancer Poh et al. (2015); Luo et al. (2023); Zeng et al. (2024)

Protein classification: Transferase; Kinase; Tyrosine-protein kinase

Ligands with Z-scores $<$ -2.58: CBC, CBD, CBDA, CBDV, CBDVA, CBG, CBGA, CBLA, CBN, THCA, THCV, THCVA, Δ-9-THC

Primary pocket Drug Score: 0.81

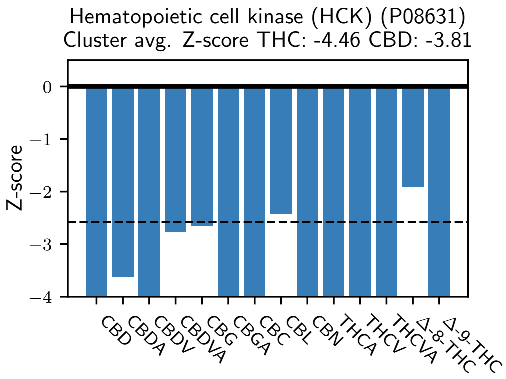

Figure S5. Z-scores of cannabinoids with Hematopoietic cell kinase (HCK) (P08631).

Table S3: Reactome pathways for Hematopoietic cell kinase (HCK) (P08631)

Reactome Pathway ID	Pathway Name	Reactome Pathway ID	Pathway Name
R-HSA-164944	Nef and signal transduction	R-HSA-9674555	Signaling by CSF3 (G-CSF)
R-HSA-2029481	FCGR activation	R-HSA-9680350	Signaling by CSF1 (M-CSF) in myeloid cells
R-HSA-912631	Regulation of signaling by CBL	R-HSA-9705462	Inactivation of CSF3 (G-CSF) signaling
R-HSA-9664323	FCGR3A-mediated IL10 synthesis	R-HSA-9706374	FLT3 signaling through SRC family kinases
R-HSA-9664422	FCGR3A-mediated phagocytosis		

5.2 GTPase KRas

Protein UniProt ID: P01116

Protein PDB ID-Chain: 5V6V-A

Membrane bound protein with GTPase activity, controls downstream pathways including cell growth, differentiation, and survival. Mutations present in $\approx 25\%$ of human cancers Uprety and Adjei (2020).

Connection with disease: Cancer Uprety and Adjei (2020)

Protein classification: Hydrolase

Ligands with Z-scores $<$ -2.58: CBC, CBDA, CBDV, CBDVA, CBG, CBL, CBLA, CBN, THCA, THCV, THCVA, Δ-8-THC, Δ-9-THC

Primary pocket Drug Score: 0.8

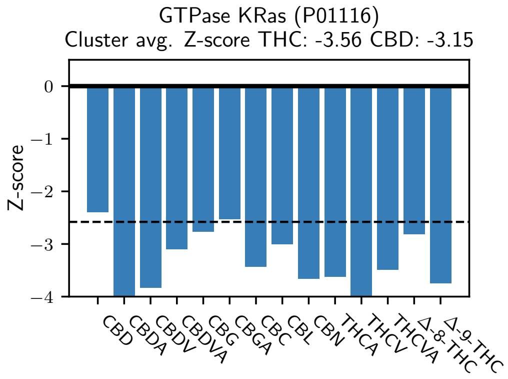

Figure S6. Z-scores of cannabinoids with GTPase KRas (P01116).

Table S4: Reactome pathways for GTPase KRas (P01116)

Reactome Pathway ID	Pathway Name	Reactome Pathway ID	Pathway Name
R-HSA-112412	SOS-mediated signalling	R-HSA-5673000	RAF activation
R-HSA-1169092	Activation of RAS in B cells	R-HSA-5673001	RAF/MAP kinase cascade
R-HSA-1236382	Constitutive Signaling by Ligand-Responsive EGFR Cancer Variants	R-HSA-5674135	MAP2K and MAPK activation
R-HSA-1250196	SHC1 events in ERBB2 signaling	R-HSA-5675221	Negative regulation of MAPK pathway
R-HSA-1250347	SHC1 events in ERBB4 signaling	R-HSA-6802946	Signaling by moderate kinase activity BRAF mutants

Continued on next page

Table S4: Reactome pathways for GTPase KRas (P01116)

Reactome Pathway ID	Pathway Name	Reactome Pathway ID	Pathway Name
R-HSA-1433557	Signaling by SCF-KIT	R-HSA-6802948	Signaling by high-kinase activity BRAF mutants
R-HSA-167044	Signalling to RAS	R-HSA-6802952	Signaling by BRAF and RAF1 fusions
R-HSA-171007	p38MAPK events	R-HSA-6802953	RAS signaling downstream of NF1 loss-of-function variants
R-HSA-179812	GRB2 events in EGFR signaling	R-HSA-6802955	Paradoxical activation of RAF signaling by kinase inactive BRAF
R-HSA-180336	SHC1 events in EGFR signaling	R-HSA-74751	Insulin receptor signalling cascade
R-HSA-186763	Downstream signal transduction	R-HSA-8849471	PTK6 Regulates RHO GTPases, RAS GTPase and MAP kinases
R-HSA-1963640	GRB2 events in ERBB2 signaling	R-HSA-8851805	MET activates RAS signaling
R-HSA-210993	Tie2 Signaling	R-HSA-8951936	RUNX3 regulates p14-ARF
R-HSA-2179392	EGFR Transactivation by Gastrin	R-HSA-9026519	Activated NTRK2 signals through RAS
R-HSA-2424491	DAP12 signaling	R-HSA-9027284	Erythropoietin activates RAS
R-HSA-2428933	SHC-related events triggered by IGF1R	R-HSA-9028731	Activated NTRK2 signals through FRS2 and FRS3
R-HSA-2871796	FCERI mediated MAPK activation	R-HSA-9034864	Activated NTRK3 signals through RAS
R-HSA-375165	NCAM signaling for neurite out-growth	R-HSA-9607240	FLT3 Signaling
R-HSA-4086398	Ca2+ pathway	R-HSA-9634285	Constitutive Signaling by Overexpressed ERBB2
R-HSA-442982	Ras activation upon Ca2+ influx through NMDA receptor	R-HSA-9634635	Estrogen-stimulated signaling through PRKCZ
R-HSA-5218921	VEGFR2 mediated cell proliferation	R-HSA-9648002	RAS processing
R-HSA-5621575	CD209 (DC-SIGN) signaling	R-HSA-9649948	Signaling downstream of RAS mutants
R-HSA-5637810	Constitutive Signaling by EGFRvIII	R-HSA-9656223	Signaling by RAF1 mutants
R-HSA-5654688	SHC-mediated cascade:FGFR1	R-HSA-9664565	Signaling by ERBB2 KD Mutants
R-HSA-5654693	FRS-mediated FGFR1 signaling	R-HSA-9665348	Signaling by ERBB2 ECD mutants
R-HSA-5654699	SHC-mediated cascade:FGFR2	R-HSA-9665686	Signaling by ERBB2 TMD/JMD mutants
R-HSA-5654700	FRS-mediated FGFR2 signaling	R-HSA-9670439	Signaling by phosphorylated juxtamembrane, extracellular and kinase domain KIT mutants
R-HSA-5654704	SHC-mediated cascade:FGFR3	R-HSA-9673767	Signaling by PDGFRA transmembrane, juxtamembrane and kinase domain mutants

Continued on next page

Table S4: Reactome pathways for GTPase KRas (P01116)

Reactome Pathway ID	Pathway Name	Reactome Pathway ID	Pathway Name
R-HSA-5654706	FRS-mediated FGFR3 signaling	R-HSA-9673770	Signaling by PDGFRA extracellular domain mutants
R-HSA-5654712	FRS-mediated FGFR4 signaling	R-HSA-9674555	Signaling by CSF3 (G-CSF)
R-HSA-5654719	SHC-mediated cascade:FGFR4	R-HSA-9680350	Signaling by CSF1 (M-CSF) in myeloid cells
R-HSA-5655253	Signaling by FGFR2 in disease	R-HSA-9703465	Signaling by FLT3 fusion proteins
R-HSA-5655291	Signaling by FGFR4 in disease	R-HSA-9703648	Signaling by FLT3 ITD and TKD mutants
R-HSA-5655302	Signaling by FGFR1 in disease	R-HSA-9753510	Signaling by RAS GAP mutants
R-HSA-5655332	Signaling by FGFR3 in disease	R-HSA-9753512	Signaling by RAS GTPase mutants
R-HSA-5658442	Regulation of RAS by GAPs		

5.3 Serine/threonine-protein kinase pim-1

Protein UniProt ID: P11309

Protein PDB ID-Chain: 3VBX-A

Serine/threonine kinase involved in cell growth, proliferation, differentiation, migration. Highly expressed in stem and cancer cells Zhao et al. (2022).

Connection with disease: Cancer Tursynbay et al. (2016)

Protein classification: Transferase; Kinase; Serine/threonine-protein kinase

Ligands with Z-scores $<$ -2.58: CBC, CBDV, CBG, CBN, Δ-8-THC

Primary pocket Drug Score: 0.8

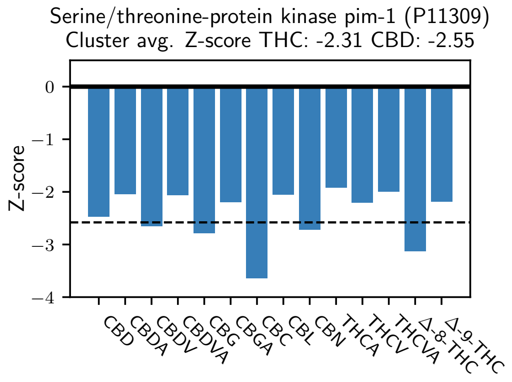

Figure S7. Z-scores of cannabinoids with Serine/threonine-protein kinase pim-1 (P11309).

Table S5: Reactome pathways for Serine/threonine-protein kinase pim-1 (P11309)

Reactome Pathway ID	Pathway Name	Reactome Pathway ID	Pathway Name
R-HSA-6785807	Interleukin-4 and Interleukin-13 signaling	R-HSA-9703465	Signaling by FLT3 fusion proteins
R-HSA-9702518	STAT5 activation downstream of FLT3 ITD mutants		

5.4 Ubiquitin-conjugating enzyme E2 N

Protein UniProt ID: P61088

Protein PDB ID-Chain: 3HCT-B

Modulates the polyubiquitination of various substrates. Upregulated in various tumor tissues Bui et al. (2021); Du et al. (2021).

Connection with disease: Cancer Bui et al. (2021); Du et al. (2021)

Protein classification: Transferase; Acyltransferase

Ligands with Z-scores $<$ -2.58: CBC, CBD, CBDV, CBL, Δ-8-THC

Primary pocket Drug Score: 0.72

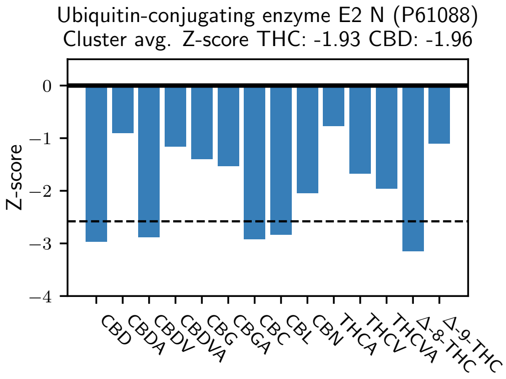

Figure S8. Z-scores of cannabinoids with Ubiquitin-conjugating enzyme E2 N (P61088).

Table S6: Reactome pathways for Ubiquitin-conjugating enzyme E2 N (P61088)

Reactome Pathway ID	Pathway Name	Reactome Pathway ID	Pathway Name
R-HSA-1169408	ISG15 antiviral mechanism	R-HSA-5693607	Processing of DNA double-strand break ends

Continued on next page

Table S6: Reactome pathways for Ubiquitin-conjugating enzyme E2 N (P61088)

Reactome Pathway ID	Pathway Name	Reactome Pathway ID	Pathway Name
R-HSA-168638	NOD1/2 Signaling Pathway	R-HSA-5696395	Formation of Incision Complex in GG-NER
R-HSA-168927	TICAM1, RIP1-mediated IKK complex recruitment	R-HSA-69473	G2/M DNA damage checkpoint
R-HSA-202424	Downstream TCR signaling	R-HSA-8866654	E3 ubiquitin ligases ubiquitinate target proteins
R-HSA-2871837	FCERI mediated NF-kB activation	R-HSA-9020702	Interleukin-1 signaling
R-HSA-445989	TAK1-dependent IKK and NF-kappa-B activation	R-HSA-937039	IRAK1 recruits IKK complex
R-HSA-450302	activated TAK1 mediates p38 MAPK activation	R-HSA-937041	IKK complex recruitment mediated by RIP1
R-HSA-450321	JNK (c-Jun kinases) phosphorylation and activation mediated by activated human TAK1	R-HSA-9646399	Aggrephagy
R-HSA-5205685	PINK1-PRKN Mediated Mitophagy	R-HSA-9705671	SARS-CoV-2 activates/modulates innate and adaptive immune responses
R-HSA-5607764	CLEC7A (Dectin-1) signaling	R-HSA-975110	TRAF6 mediated IRF7 activation in TLR7/8 or 9 signaling
R-HSA-5693565	Recruitment and ATM-mediated phosphorylation of repair and signaling proteins at DNA double strand breaks	R-HSA-975144	IRAK1 recruits IKK complex upon TLR7/8 or 9 stimulation
R-HSA-5693571	Nonhomologous End-Joining (NHEJ)	R-HSA-983168	Antigen processing: Ubiquitination & Proteasome degradation

5.5 3',5'-cyclic-AMP phosphodiesterase 4B

Protein UniProt ID: Q07343

Protein PDB ID-Chain: 3WD9-A

Important intracellular signaling regulator, hydrolyzes the second messenger cAMP Tibbo and Baillie (2020).

Connection with disease: Cancer Kim et al. (2019), Neurological diseases Tibbo and Baillie (2020), Inflammation Blauvelt et al. (2023)

Protein classification: Hydrolase

Ligands with Z-scores $<$ -2.58: CBLA, CBN, THCA, Δ-9-THC

Primary pocket Drug Score: 0.8

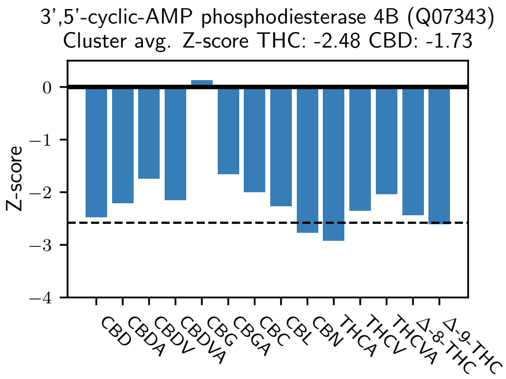

Figure S9. Z-scores of cannabinoids with 3’,5’-cyclic-AMP phosphodiesterase 4B (Q07343).

Table S7. Reactome pathways for 3’,5’-cyclic-AMP phosphodiesterase 4B (Q07343)

Reactome Pathway ID	Pathway Name
R-HSA-180024	DARPP-32 events

5.6 Cyclin-dependent kinase 2

Protein UniProt ID: P24941

Protein PDB ID-Chain: 6Q49-A

Serine/threonine kinase, whose activity depends on a cyclin, involved in cell cycle control Malumbres (2014).

Connection with disease: Cancer Tadesse et al. (2018)

Protein classification: Transferase; Kinase; Serine/threonine-protein kinase

Ligands with Z-scores $<$ -2.58: CBD, CBN, THCV

Primary pocket Drug Score: 0.81

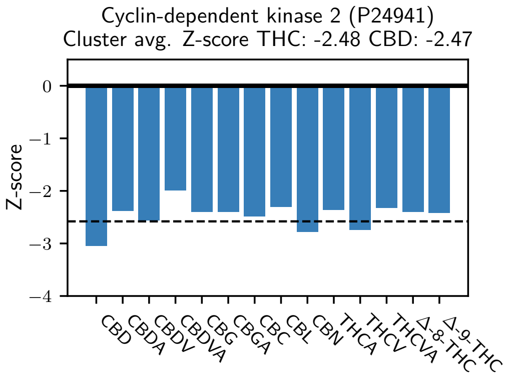

Figure S10. Z-scores of cannabinoids with Cyclin-dependent kinase 2 (P24941).

Table S8: Reactome pathways for Cyclin-dependent kinase 2 (P24941)

Reactome Pathway ID	Pathway Name	Reactome Pathway ID	Pathway Name
R-HSA-1538133	G0 and Early G1	R-HSA-69017	CDK-mediated phosphorylation and removal of Cdc6
R-HSA-171319	Telomere Extension By Telomerase	R-HSA-69200	Phosphorylation of proteins involved in G1/S transition by active Cyclin E:Cdk2 complexes
R-HSA-176187	Activation of ATR in response to replication stress	R-HSA-69202	Cyclin E associated events during G1/S transition
R-HSA-176408	Regulation of APC/C activators between G1/S and early anaphase	R-HSA-69231	Cyclin D associated events in G1
R-HSA-187577	SCF(Skp2)-mediated degradation of p27/p21	R-HSA-69273	Cyclin A/B1/B2 associated events during G2/M transition
R-HSA-2559582	Senescence-Associated Secretory Phenotype (SASP)	R-HSA-69563	p53-Dependent G1 DNA Damage Response
R-HSA-2559586	DNA Damage/Telomere Stress Induced Senescence	R-HSA-69656	Cyclin A:Cdk2-associated events at S phase entry
R-HSA-5693607	Processing of DNA double-strand break ends	R-HSA-8849470	PTK6 Regulates Cell Cycle
R-HSA-6804116	TP53 Regulates Transcription of Genes Involved in G1 Cell Cycle Arrest	R-HSA-912446	Meiotic recombination
R-HSA-6804756	Regulation of TP53 Activity through Phosphorylation	R-HSA-9616222	Transcriptional regulation of granulopoiesis
R-HSA-6804757	Regulation of TP53 Degradation	R-HSA-9661069	Defective binding of RB1 mutants to E2F1,(E2F2, E2F3)
R-HSA-68911	G2 Phase	R-HSA-9825892	Regulation of MITF-M-dependent genes involved in cell cycle and proliferation
R-HSA-68949	Orc1 removal from chromatin	R-HSA-983231	Factors involved in megakaryocyte development and platelet production
R-HSA-68962	Activation of the pre-replicative complex		

5.7 Estrogen receptor

Protein UniProt ID: P03372

Protein PDB ID-Chain: 4IVY-A

Nuclear hormone receptor involved in gene expression regulation as well as cell proliferation and differentiation. Expressed in around 75% of breast cancers Grinshpun et al. (2023).

Connection with disease: (Breast) Cancer Grinshpun et al. (2023)

Protein classification: Receptor

Ligands with Z-scores < -2.58: CBDA, CBL, CBN, Δ-8-THC, Δ-9-THC

Primary pocket Drug Score: 0.81

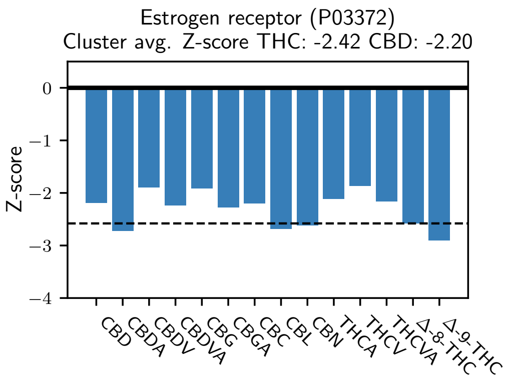

Figure S11. Z-scores of cannabinoids with Estrogen receptor (P03372).

Table S9: Reactome pathways for Estrogen receptor (P03372)

Reactome Pathway ID	Pathway Name	Reactome Pathway ID	Pathway Name
R-HSA-1251985	Nuclear signaling by ERBB4	R-HSA-8931987	RUNX1 regulates estrogen receptor mediated transcription
R-HSA-1257604	PIP3 activates AKT signaling	R-HSA-8939211	ESR-mediated signaling
R-HSA-2219530	Constitutive Signaling by Aberrant PI3K in Cancer	R-HSA-8939256	RUNX1 regulates transcription of genes involved in WNT signaling
R-HSA-383280	Nuclear Receptor transcription pathway	R-HSA-8939902	Regulation of RUNX2 expression and activity
R-HSA-4090294	SUMOylation of intracellular receptors	R-HSA-9009391	Extra-nuclear estrogen signaling
R-HSA-5689896	Ovarian tumor domain proteases	R-HSA-9018519	Estrogen-dependent gene expression
R-HSA-6811558	PI5P, PP2A and IER3 Regulate PI3K/AKT Signaling	R-HSA-9841251	Mitochondrial unfolded protein response (UPRmt)
R-HSA-8866910	TFAP2 (AP-2) family regulates transcription of growth factors and their receptors		

5.8 Histone-lysine N-methyltransferase EHMT1

Protein UniProt ID: Q9H9B1

Protein PDB ID-Chain: 3MO0-A

Methylates lysines of histone H3 alongside other substrates. Plays a role in embryo development, tumor cell growth and metastasis as well as cognition Milite et al. (2019).

Connection with disease: Cancer Huang et al. (2010), Neurological diseases Zheng et al. (2019)

Protein classification: Transferase; Methyltransferase

Ligands with Z-scores < -2.58: THCA, THCVA, Δ-9-THC

Primary pocket Drug Score: 0.8

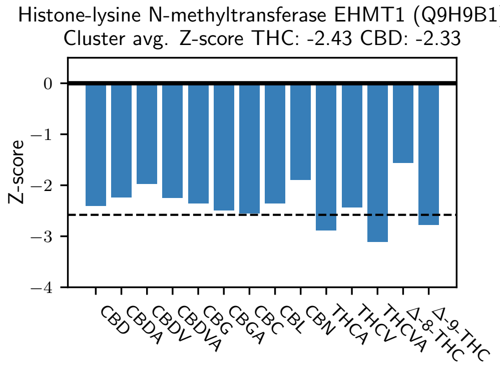

Figure S12. Z-scores of cannabinoids with Histone-lysine N-methyltransferase EHMT1 (Q9H9B1).

Table S10: Reactome pathways for Histone-lysine N-methyltransferase EHMT1 (Q9H9B1)

Reactome Pathway ID	Pathway Name	Reactome Pathway ID	Pathway Name
R-HSA-2559582	Senescence-Associated Secretory Phenotype (SASP)	R-HSA-8853884	Transcriptional Regulation by VENTX
R-HSA-3214841	PKMTs methylate histone lysines	R-HSA-8953750	Transcriptional Regulation by E2F6
R-HSA-6804760	Regulation of TP53 Activity through Methylation	R-HSA-9843970	Regulation of endogenous retroelements by the Human Silencing Hub (HUSH) complex

5.9 Histone-arginine methyltransferase CARM1

Protein UniProt ID: Q86X55

Protein PDB ID-Chain: 6S7C-A

Methylates arginines of histone H3 alongside other substrates. Upregulated in many types of human cancer Jin et al. (2023).

Connection with disease: Cancer Jin et al. (2023)

Protein classification: Transferase; Methyltransferase

Ligands with Z-scores < -2.58: CBD, CBDA, CBDVA

Primary pocket Drug Score: 0.81

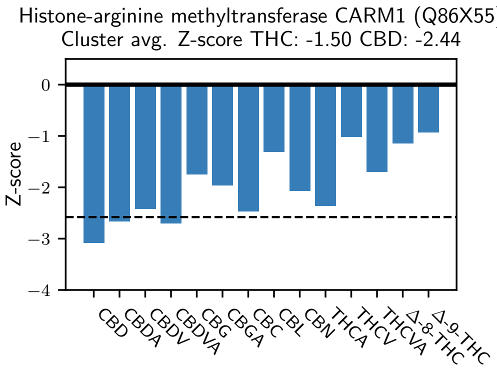

Figure S13. Z-scores of cannabinoids with Histone-arginine methyltransferase CARM1 (Q86X55).

Table S11: Reactome pathways for Histone-arginine methyltransferase CARM1 (Q86X55)

Reactome Pathway ID	Pathway Name	Reactome Pathway ID	Pathway Name
R-HSA-1368082	RORA activates gene expression	R-HSA-400206	Regulation of lipid metabolism by PPARalpha
R-HSA-1368108	BMAL1:CLOCK,NPAS2 activates circadian gene expression	R-HSA-400253	Circadian Clock
R-HSA-1989781	PPARA activates gene expression	R-HSA-6804114	TP53 Regulates Transcription of Genes Involved in G2 Cell Cycle Arrest
R-HSA-2151201	Transcriptional activation of mitochondrial biogenesis	R-HSA-9018519	Estrogen-dependent gene expression
R-HSA-2426168	Activation of gene expression by SREBF (SREBP)	R-HSA-9707564	Cytoprotection by HMOX1
R-HSA-3214858	RMTs methylate histone arginines	R-HSA-9707616	Heme signaling
R-HSA-381340	Transcriptional regulation of white adipocyte differentiation		

5.10 N-lysine methyltransferase KMT5A

Protein UniProt ID: Q9NQR1

Protein PDB ID-Chain: 5TH7-A

Methylates lysines of histone H4 alongside other substrates. Regulates cell cycle progression and helps maintain genome integrity Lin et al. (2019).

Connection with disease: Cancer Lin et al. (2019)

Protein classification: Transferase; Methyltransferase

Ligands with Z-scores $<$ -2.58: CBGA, CBN, THCV

Primary pocket Drug Score: 0.78

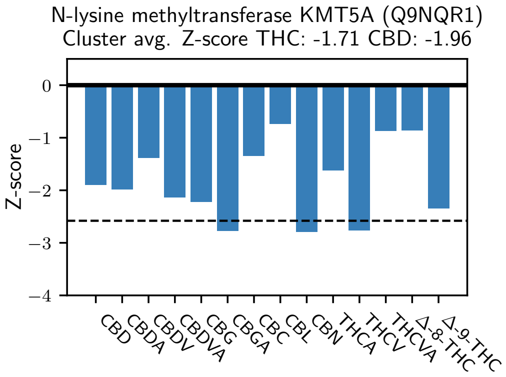

Figure S14. Z-scores of cannabinoids with N-lysine methyltransferase KMT5A (Q9NQR1).

Table S12: Reactome pathways for N-lysine methyltransferase KMT5A (Q9NQR1)

Reactome Pathway ID	Pathway Name	Reactome Pathway ID	Pathway Name
R-HSA-2299718	Condensation of Prophase Chromosomes	R-HSA-6804760	Regulation of TP53 Activity through Methylation
R-HSA-3214841	PKMTs methylate histone lysines		

5.11 Pyruvate kinase PKM, isozyme M2

Protein UniProt ID: P14618

Protein PDB ID-Chain: 3BJT-B

Controls the rate-limiting step of glycolysis, regulated by intercellular signaling Israelsen and Vander Heiden (2015).

Connection with disease: Cancer Israelsen and Vander Heiden (2015)

Protein classification: Transferase; Kinase

Ligands with Z-scores $<$ -2.58: CBDA, CBDVA, CBL, THCA

Primary pocket Drug Score: 0.81

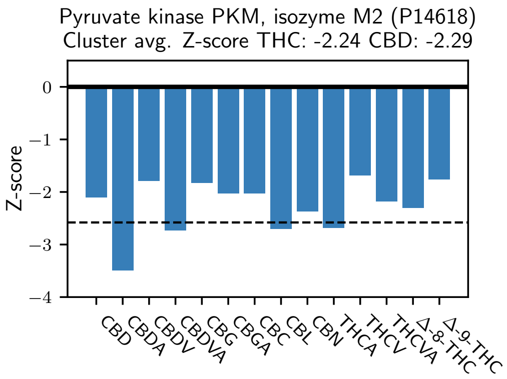

Figure S15. Z-scores of cannabinoids with Pyruvate kinase PKM, isozyme M2 (P14618).

Table S13: Reactome pathways for Pyruvate kinase PKM, isozyme M2 (P14618)

Reactome Pathway ID	Pathway Name	Reactome Pathway ID	Pathway Name
R-HSA-6798695	Neutrophil degranulation	R-HSA-70268	Pyruvate metabolism
R-HSA-70171	Glycolysis	R-HSA-9861718	Regulation of pyruvate metabolism

5.12 Glutaminase kidney isoform, mitochondrial

Protein UniProt ID: O94925

Protein PDB ID-Chain: 3VP3-A

Catalyses the first reaction of the primary pathway for the renal catabolism of glutamine. Upregulated in several types of cancer Masisi et al. (2020).

Connection with disease: Cancer Masisi et al. (2020)

Protein classification: Hydrolase

Ligands with Z-scores < -2.58: CBD, CBDV, CBG, CBGA, CBN

Primary pocket Drug Score: 0.79

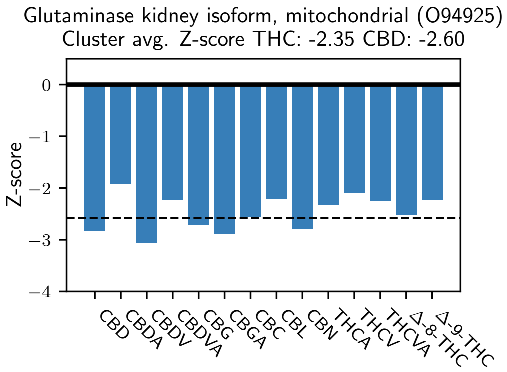

Figure S16. Z-scores of cannabinoids with Glutaminase kidney isoform, mitochondrial (O94925).

Table S14: Reactome pathways for Glutaminase kidney isoform, mitochondrial (O94925)

5.13 Glycogen phosphorylase, liver form

Protein UniProt ID: P06737

Protein PDB ID-Chain: 1EM6-A

Rate-limiting enzyme for renal glycogen catabolism, regulates blood glucose concentration He et al. (2023).

Connection with disease: Cancer He et al. (2023); Zois et al. (2022)

Protein classification: Transferase; Glycosyltransferase

Ligands with Z-scores < -2.58: CBD, CBDA, CBDVA, CBN

Primary pocket Drug Score: 0.81

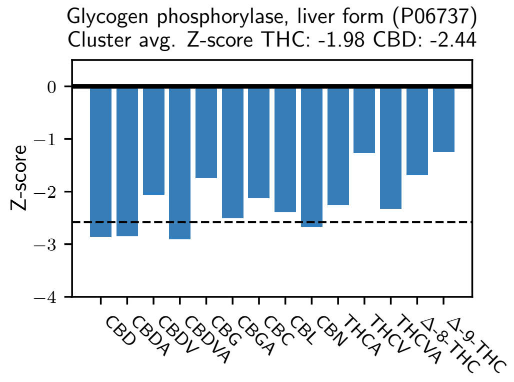

Figure S17. Z-scores of cannabinoids with Glycogen phosphorylase, liver form (P06737).

Table S15: Reactome pathways for Glycogen phosphorylase, liver form (P06737)

Reactome Pathway ID	Pathway Name	Reactome Pathway ID	Pathway Name
R-HSA-6798695	Neutrophil degranulation	R-HSA-70221	Glycogen breakdown (glycogenolysis)

5.14 Fructose-1,6-bisphosphatase 1

Protein UniProt ID: P09467

Protein PDB ID-Chain: 2Y5K-C

Rate-limiting enzyme in gluconeogenesis, catalyses hydrolysis of fructose 1,6-bisphosphate. Reduced activity reported in some types of cancer. Inhibition suggested as a potential therapy of type II diabetes Timson (2019).

Connection with disease: Diabetes Kaur et al. (2017), Cancer Lu et al. (2020)

Protein classification: Hydrolase

Ligands with Z-scores < -2.58: CBN, THCV, THCVA

Primary pocket Drug Score: 0.84

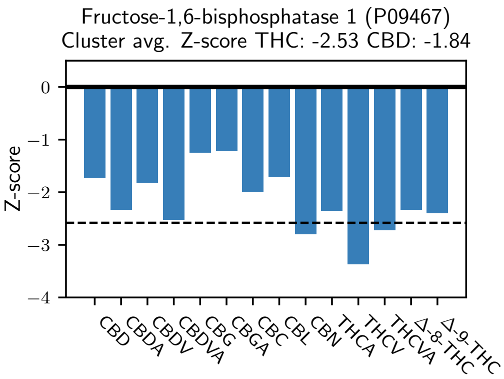

Figure S18. Z-scores of cannabinoids with Fructose-1,6-bisphosphatase 1 (P09467).

Table S16. Reactome pathways for Fructose-1,6-bisphosphatase 1 (P09467)

Reactome Pathway ID	Pathway Name
R-HSA-70263	Gluconeogenesis

5.15 MMP1 – Interstitial collagenase

Protein UniProt ID: P03956

Protein PDB ID-Chain: 2AYK-A

Matrix metalloproteinase, cleaves collagens of types I, II, and III. Expressed in various cancer tissues Pytliak et al. (2012).

Connection with disease: Cancer Pytliak et al. (2012), Arthritis Wang et al. (2020)

Protein classification: Hydrolase; Protease; Metalloprotease

Ligands with Z-scores < -2.58: CBC, CBDA, CBN, THCA, THCV, THCVA, Δ-8-THC, Δ-9-THC

Primary pocket Drug Score: 0.81

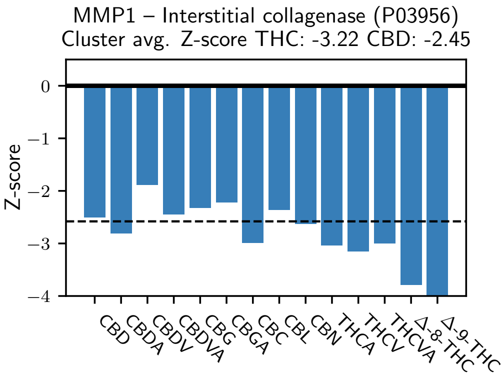

Figure S19. Z-scores of cannabinoids with MMP1 – Interstitial collagenase (P03956).

Table S17: Reactome pathways for MMP1 – Interstitial collagenase (P03956)

Reactome Pathway ID	Pathway Name	Reactome Pathway ID	Pathway Name
R-HSA-1442490	Collagen degradation	R-HSA-210991	Basigin interactions
R-HSA-1474228	Degradation of the extracellular matrix	R-HSA-381426	Regulation of Insulin-like Growth Factor (IGF) transport and uptake by Insulin-like Growth Factor Binding Proteins (IGFBPs)
R-HSA-1592389	Activation of Matrix Metalloproteinases	R-HSA-6785807	Interleukin-4 and Interleukin-13 signaling

5.16 MMP13 – Collagenase 3

Protein UniProt ID: P45452

Protein PDB ID-Chain: 1FLS-A

Matrix metalloproteinase, plays a role in extracellular matrix degradation. Exhibits the highest activity with soluble type II collagen, plays a role in cartilage for degradation Wang et al. (2013).

Connection with disease: Cancer Pytliak et al. (2012), Arthritis Wang et al. (2013)

Protein classification: Hydrolase; Protease; Metalloprotease

Ligands with Z-scores $<$ -2.58: CBC, CBD, CBDV, CBG, CBGA, CBL, THCV, Δ-8-THC

Primary pocket Drug Score: 0.84

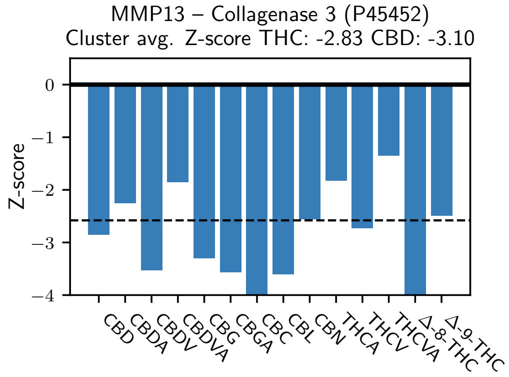

Figure S20. Z-scores of cannabinoids with MMP13 – Collagenase 3 (P45452).

Table S18: Reactome pathways for MMP13 – Collagenase 3 (P45452)

Reactome Pathway ID	Pathway Name	Reactome Pathway ID	Pathway Name
R-HSA-1442490	Collagen degradation	R-HSA-2022090	Assembly of collagen fibrils and other multimeric structures
R-HSA-1474228	Degradation of the extracellular matrix	R-HSA-8941332	RUNX2 regulates genes involved in cell migration
R-HSA-1592389	Activation of Matrix Metalloproteinases		

5.17 MMP3 – Stromelysin-1

Protein UniProt ID: P08254

Protein PDB ID-Chain: 2JT5-A

Matrix metalloproteinase with broad substrate specificity, degrades collagen types II, III, IV, IX and X, proteoglycans, fibronectin, laminin, and elastin. Activates other molecules, including MMPs Lerner et al. (2018).

Connection with disease: Cancer Pytliak et al. (2012), Arthritis Lerner et al. (2018)

Protein classification: Hydrolase; Protease; Metalloprotease

Ligands with Z-scores $<$ -2.58: CBC, CBD, CBDV, CBDVA, CBG, CBL, CBLA

Primary pocket Drug Score: 0.75

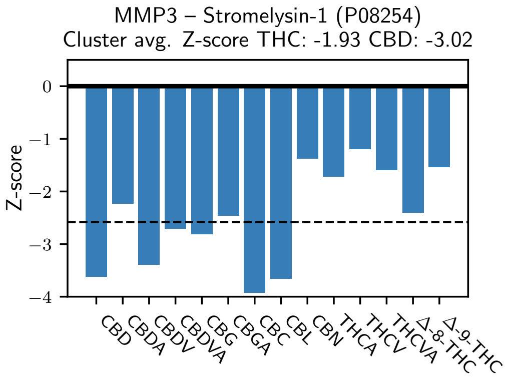

Figure S21. Z-scores of cannabinoids with MMP3 – Stromelysin-1 (P08254).

Table S19: Reactome pathways for MMP3 – Stromelysin-1 (P08254)

Reactome Pathway ID	Pathway Name	Reactome Pathway ID	Pathway Name
R-HSA-1442490	Collagen degradation	R-HSA-2179392	EGFR Transactivation by Gastrin
R-HSA-1474228	Degradation of the extracellular matrix	R-HSA-6785807	Interleukin-4 and Interleukin-13 signaling
R-HSA-1592389	Activation of Matrix Metalloproteinases	R-HSA-9009391	Extra-nuclear estrogen signaling
R-HSA-2022090	Assembly of collagen fibrils and other multimeric structures		

5.18 MMP9 – Matrix metalloproteinase-9

Protein UniProt ID: P14780

Protein PDB ID-Chain: 2OVX-A

Matrix metalloproteinase, plays an important role in the degradation of collagen type IV and gelatin Pytliak et al. (2012).

Connection with disease: Cancer Pytliak et al. (2012), Arthritis He et al. (2018)

Protein classification: Hydrolase; Protease; Metalloprotease

Ligands with Z-scores < -2.58: CBC, CBL, Δ-8-THC

Primary pocket Drug Score: 0.81

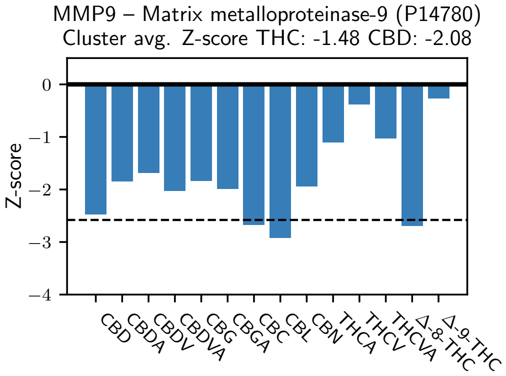

Figure S22. Z-scores of cannabinoids with MMP9 – Matrix metalloproteinase-9 (P14780).

Table S20: Reactome pathways for MMP9 – Matrix metalloproteinase-9 (P14780)

Reactome Pathway ID	Pathway Name	Reactome Pathway ID	Pathway Name
R-HSA-1433557	Signaling by SCF-KIT	R-HSA-3928665	EPH-ephrin mediated repulsion of cells
R-HSA-1442490	Collagen degradation	R-HSA-6785807	Interleukin-4 and Interleukin-13 signaling
R-HSA-1474228	Degradation of the extracellular matrix	R-HSA-6798695	Neutrophil degranulation
R-HSA-1592389	Activation of Matrix Metalloproteinases	R-HSA-9009391	Extra-nuclear estrogen signaling
R-HSA-2022090	Assembly of collagen fibrils and other multimeric structures		

5.19 E-cadherin

Protein UniProt ID: P12830

Protein PDB ID-Chain: 4ZTE-B

Cell adhesion protein that plays a key role in controlling epithelial cell adhesion, movement, and proliferation. Its dysfunction is linked to enhanced cancer progression through increased cell proliferation, invasion, and metastasis Zeng et al. (2015)

Connection with disease: Cancer Zeng et al. (2015)

Protein classification: Cell adhesion

Ligands with Z-scores < -2.58: CBD, CBDV, CBG

Primary pocket Drug Score: 0.73

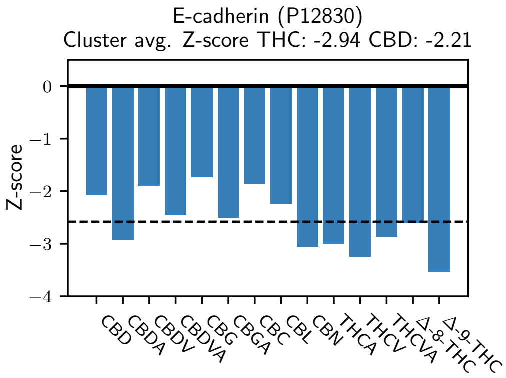

Figure S23. Z-scores of cannabinoids with E-cadherin (P12830).

Table S21: Reactome pathways for E-cadherin (P12830)

Reactome Pathway ID	Pathway Name	Reactome Pathway ID	Pathway Name
R-HSA-1474228	Degradation of the extracellular matrix	R-HSA-5626467	RHO GTPases activate IQGAPs
R-HSA-198933	Immunoregulatory interactions between a Lymphoid and a non-Lymphoid cell	R-HSA-8876493	InlA-mediated entry of Listeria monocytogenes into host cells
R-HSA-216083	Integrin cell surface interactions	R-HSA-9823730	Formation of definitive endoderm
R-HSA-351906	Apoptotic cleavage of cell adhesion proteins	R-HSA-9856649	Transcriptional and post-translational regulation of MITF-M expression and activity
R-HSA-418990	Adherens junctions interactions	R-HSA-9926550	Regulation of MITF-M-dependent genes involved in extracellular matrix, focal adhesion and epithelial-to-mesenchymal transition

5.20 MMP12 – Macrophage metalloelastase

Protein UniProt ID: P39900

Protein PDB ID-Chain: 3F16-A

Matrix metalloproteinase, primarily known for elastolytic activity, capable of degrading a broad spectrum of other extracellular matrix components.

Connection with disease: Inflammatory diseases Lagente et al. (2009), Neurological diseases Chelluboina et al. (2018)

Protein classification: Hydrolase; Protease; Metalloprotease

Ligands with Z-scores $<$ -2.58: CBC, CBGA, CBN, THCV, Δ-8-THC, Δ-9-THC

Primary pocket Drug Score: 0.74

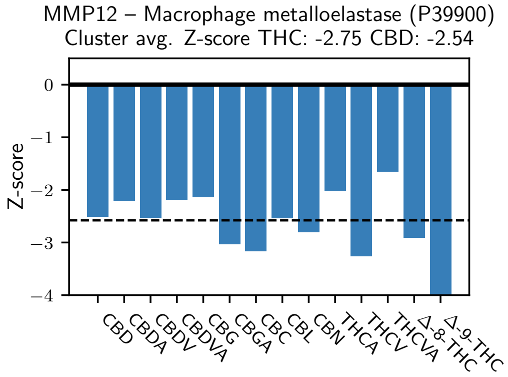

Figure S24. Z-scores of cannabinoids with MMP12 – Macrophage metalloelastase (P39900).

Table S22: Reactome pathways for MMP12 – Macrophage metalloelastase (P39900)

Reactome Pathway ID	Pathway Name	Reactome Pathway ID	Pathway Name
R-HSA-1442490	Collagen degradation	R-HSA-1474228	Degradation of the extracellular matrix

5.21 Amine oxidase [flavin-containing] B

Protein UniProt ID: P27338

Protein PDB ID-Chain: 6FVZ-B

Catalyses oxidation of monoamines at the outer mitochondrial membrane, mediates GABA synthesis. Expression significantly increased in neuroinflammatory and neurodegenerative conditions Nam et al. (2022).

Connection with disease: Parkinson's disease Nam et al. (2022), Alzheimer's disease Nam et al. (2024)

Protein classification: Oxidoreductase

Ligands with Z-scores $<$ -2.58: CBD, CBDA, CBDVA, CBG, THCV, Δ-9-THC

Primary pocket Drug Score: 0.81

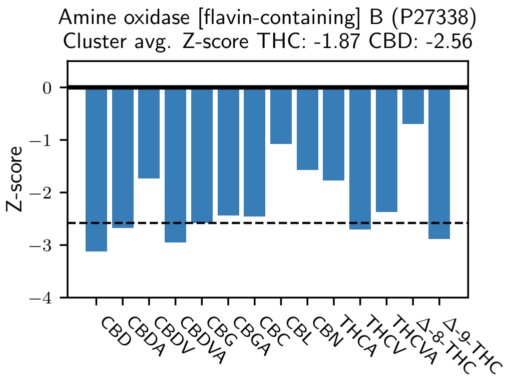

Figure S25. Z-scores of cannabinoids with Amine oxidase [flavin-containing] B (P27338).

Table S23. Reactome pathways for Amine oxidase [flavin-containing] B (P27338)

Reactome Pathway ID	Pathway Name
R-HSA-141333	Biogenic amines are oxidatively deaminated to aldehydes by MAOA and MAOB

5.22 Beta-secretase 1

Protein UniProt ID: P56817

Protein PDB ID-Chain: 2QZK-A

Aspartyl protease involved in the formation of myelin sheaths. Cleaves amyloid precursor protein, generates amyloid-β peptides which accumulate in brains with Alzheimer's disease Moussa (2017).

Connection with disease: Alzheimer's disease Moussa (2017)

Protein classification: Hydrolase; Protease; Aspartyl protease

Ligands with Z-scores $<$ -2.58: CBD, CBDV, CBG, CBL, THCA

Primary pocket Drug Score: 0.8

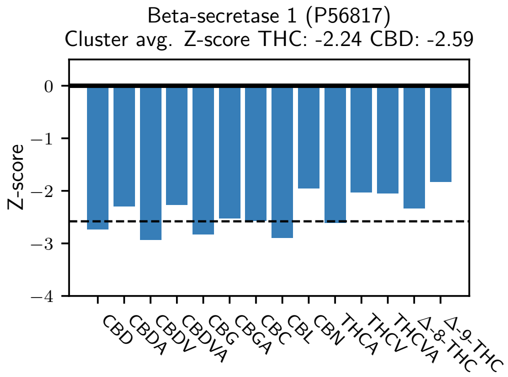

Figure S26. Z-scores of cannabinoids with Beta-secretase 1 (P56817).

Table S24. Reactome pathways for Beta-secretase 1 (P56817)

Reactome Pathway ID	Pathway Name
R-HSA-977225	Amyloid fiber formation

5.23 Coagulation factor X

Protein UniProt ID: P00742

Protein PDB ID-Chain: 2D1J-A

Key driver of thrombin generation, central to the coagulation system. Regulation of active FXa form has a major impact on blood clotting Camire (2021).

Connection with disease: Blood coagulation Camire (2021)

Protein classification: Hydrolase; Protease; Serine protease

Ligands with Z-scores $<$ -2.58: CBDV, CBDVA, CBN, THCA, THCV, THCVA, Δ-8-THC

Primary pocket Drug Score: 0.74

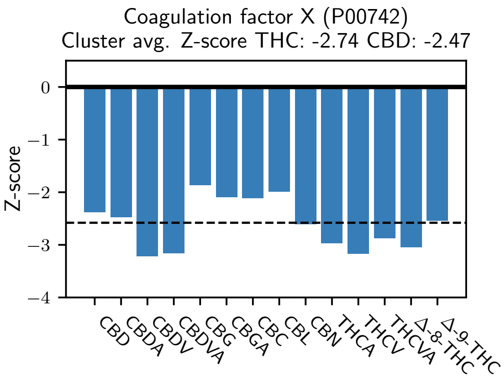

Figure S27. Z-scores of cannabinoids with Coagulation factor X (P00742).

Table S25: Reactome pathways for Coagulation factor X (P00742)

Reactome Pathway ID	Pathway Name	Reactome Pathway ID	Pathway Name
R-HSA-140834	Extrinsic Pathway of Fibrin Clot Formation	R-HSA-159782	Removal of aminoterminal propeptides from gamma-carboxylated proteins
R-HSA-140837	Intrinsic Pathway of Fibrin Clot Formation	R-HSA-9672383	Defective factor IX causes thrombophilia
R-HSA-140875	Common Pathway of Fibrin Clot Formation	R-HSA-9672396	Defective cofactor function of FVIIIa variant
R-HSA-159740	Gamma-carboxylation of protein precursors	R-HSA-9673202	Defective F9 variant does not activate FX
R-HSA-159763	Transport of gamma-carboxylated protein precursors from the endoplasmic reticulum to the Golgi apparatus		

5.24 Complement factor D

Protein UniProt ID: P00746

Protein PDB ID-Chain: 1DFP-B

Rate limiting part of the alternate pathway of the complement system, primarily produced by adipocytes Barratt and Weitz (2021).

Connection with disease: Complement-driven diseases Barratt and Weitz (2021)

Protein classification: Hydrolase; Protease; Serine protease

Ligands with Z-scores < -2.58: CBDVA, CBN, THCA, THCVA, Δ-9-THC

Primary pocket Drug Score: 0.8

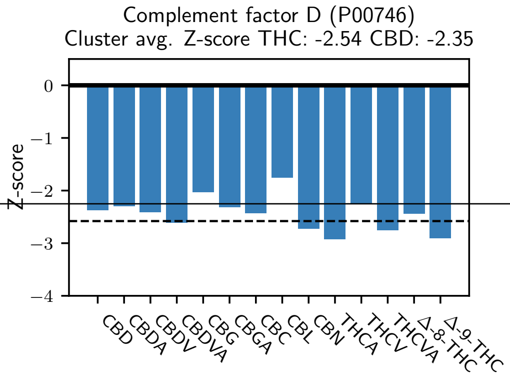

Table S26: Reactome pathways for Complement factor D (P00746)

Reactome Pathway ID	Pathway Name	Reactome Pathway ID	Pathway Name
R-HSA-114608	Platelet degranulation	R-HSA-6798695	Neutrophil degranulation
R-HSA-173736	Alternative complement activation		

6 RESULTS FOR INDIVIDUAL LIGANDS

The following sections include histograms displaying the distribution of docking scores for each cannabinoid (blue), accompanied by a plot of a normal distribution with matching mean and standard deviation (orange). The dashed red lines represent Z-scores below -2.58. Tables of the top 10 highest-scoring targets for each cannabinoid are also provided.

6.1 CBC

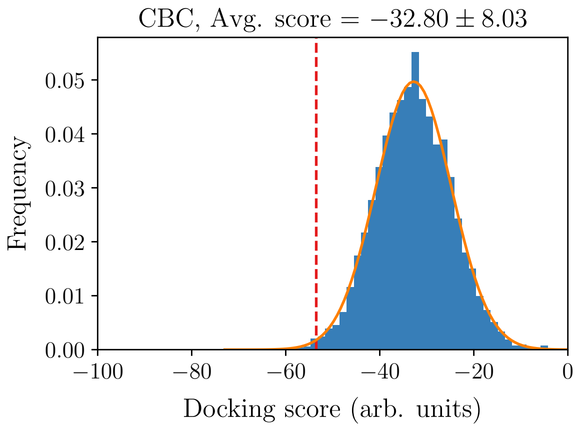

Figure S29. Histogram of docking scores for CBC

Table S27. Top 10 targets for CBC

UniProt ID	PDB ID - Chain	Protein Name	Docking Score (arb. units)	Z-Score
P08631	2C0T - A	Tyrosine-protein kinase HCK	-72.97	-6.04
P45452	1FLS - A	MMP-13, Collagenase 3	-68.23	-5.38
P08254	2JT5 - A	MMP-3, Stromelysin-1	-64.64	-4.88
P11309	3VBX - A	Serine/threonine-protein kinase pim-1	-62.37	-4.57
P01116	4LV6 - B	GTPase KRas	-60.65	-4.33
P39900	3F16 - A	MMP-12, Macrophage metalloelastase	-58.49	-4.03
P03956	2AYK - A	MMP-1, Interstitial collagenase	-57.10	-3.83
P61088	3HCT - B	Ubiquitin-conjugating enzyme E2 N	-56.53	-3.75
P61769	3GSV - B	Beta-2-microglobulin	-55.75	-3.65
O14732	2DDK - B	Inositol monophosphatase 2	-55.32	-3.59

6.2 CBD

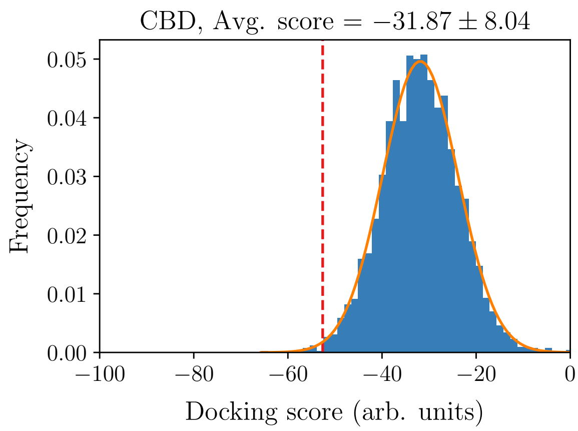

Figure S30. Histogram of docking scores for CBD

Table S28. Top 10 targets for CBD

UniProt ID	PDB ID - Chain	Protein Name	Docking Score (arb. units)	Z-Score
P08631	2C0T - A	Tyrosine-protein kinase HCK	-65.69	-5.25
P08254	2JT5 - A	MMP-3, Stromelysin-1	-61.38	-4.64
Q16658	1DFC - A	Fascin	-58.45	-4.23
P27338	6FVZ - B	Amine oxidase [flavin-containing] B	-57.28	-4.07
Q86X55	6S7C - A	Histone-arginine methyltransferase CARM1	-56.98	-4.03
P24941	6Q49 - A	Cyclin-dependent kinase 2	-56.70	-3.99
P61088	3HCT - B	Ubiquitin-conjugating enzyme E2 N	-56.01	-3.89
Q96GA7	2RKB - A	Serine dehydratase-like	-55.80	-3.86
P62937	2RMB - C	Peptidyl-prolyl cis-trans isomerase A	-55.76	-3.86
P06737	1EM6 - A	Glycogen phosphorylase, liver form	-55.20	-3.78

6.3 CBDA

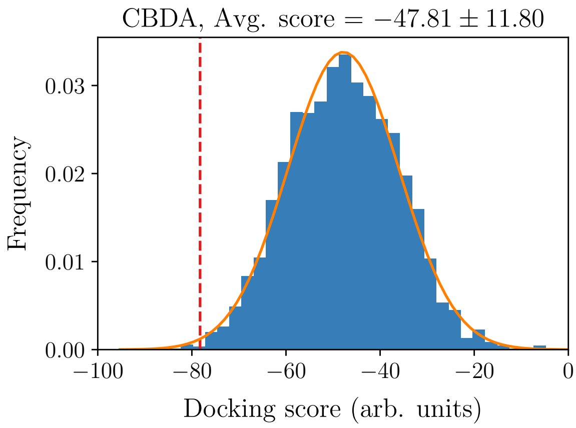

Figure S31. Histogram of docking scores for CBDA

Table S29. Top 10 targets for CBDA

UniProt ID	PDB ID - Chain	Protein Name	Docking Score (arb. units)	Z-Score
P01116	4LUC - B	GTPase KRas	-95.28	-4.96
P08631	2C0T - A	Tyrosine-protein kinase HCK	-90.99	-4.56
P14618	5X1V - B	Pyruvate kinase PKM	-89.52	-4.42
P12830	4ZT1 - A	Cadherin-1	-82.87	-3.79
P06737	3CEJ - B	Glycogen phosphorylase, liver form	-81.87	-3.69
P48735	5I95 - A	Isocitrate dehydrogenase [NADP], mitochondrial	-81.62	-3.67
P03956	2AYK - A	MMP-1, Interstitial collagenase	-81.34	-3.64
O15530	3QC4 - A	3-phosphoinositide-dependent protein kinase 1	-81.20	-3.63
P03372	4IVY - A	Estrogen receptor	-80.36	-3.55
P27338	2BYB - A	Amine oxidase [flavin-containing] B	-79.71	-3.49

6.4 CBDV

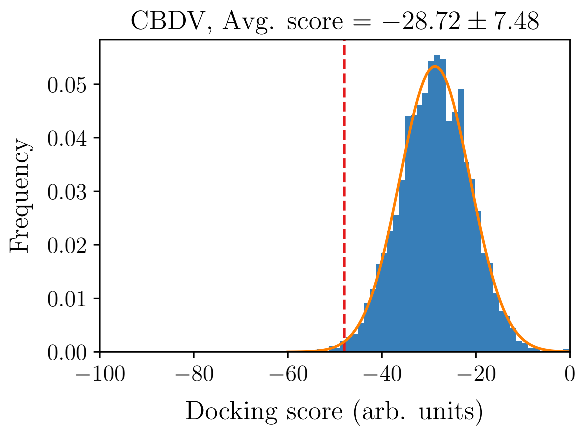

Figure S32. Histogram of docking scores for CBDV

Table S30. Top 10 targets for CBDV

UniProt ID	PDB ID - Chain	Protein Name	Docking Score (arb. units)	Z-Score
P08631	2C0T - A	Tyrosine-protein kinase HCK	-60.03	-5.28
P01116	4LUC - B	GTPase KRas	-57.76	-4.94
P45452	1FLS - A	MMP-13, Collagenase 3	-55.50	-4.59
P08254	2JT5 - A	MMP-3, Stromelysin-1	-54.43	-4.43
P48735	5I95 - A	Isocitrate dehydrogenase [NADP], mitochondrial	-53.31	-4.26
P00742	2D1J - A	Coagulation factor X	-53.13	-4.24
O94925	5JYP - A	Glutaminase kidney isoform, mitochondrial	-52.01	-4.07
Q9NRG4	6CBX - A	N-lysine methyltransferase SMYD2	-51.26	-3.95
P56817	2XFI - A	Beta-secretase 1	-51.01	-3.91
P68871	4ROL - B	Hemoglobin subunit beta	-50.74	-3.87

6.5 CBDVA

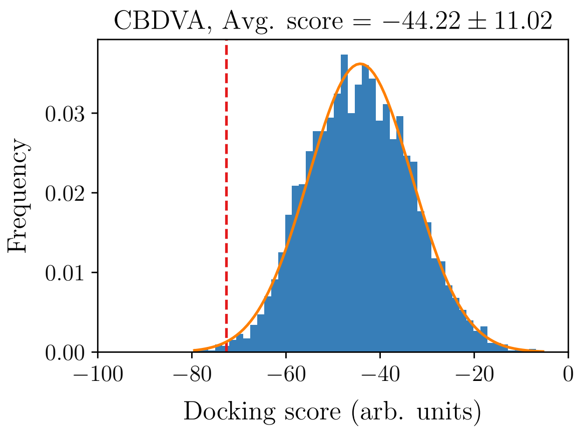

Figure S33. Histogram of docking scores for CBDVA

Table S31. Top 10 targets for CBDVA

UniProt ID	PDB ID - Chain	Protein Name	Docking Score (arb. units)	Z-Score
P00742	2D1J - A	Coagulation factor X	-79.46	-4.05
P01116	4LV6 - B	GTPase KRas	-78.78	-3.98
P27338	2BK3 - B	Amine oxidase [flavin-containing] B	-77.07	-3.81
P06737	1L5Q - B	Glycogen phosphorylase, liver form	-76.58	-3.76
P08631	2C0T - A	Tyrosine-protein kinase HCK	-75.08	-3.60
P14618	5X1W - D	Pyruvate kinase PKM	-74.69	-3.56
Q86X55	6S7A - C	Histone-arginine methyltransferase CARM1	-74.39	-3.53
P08254	1OO9 - A	MMP-3, Stromelysin-1	-74.37	-3.53
O94804	6I2Y - A	Serine/threonine-protein kinase 10	-74.25	-3.52
P55055	6S4U - A	Oxysterols receptor LXR-beta	-73.66	-3.46

6.6 CBG

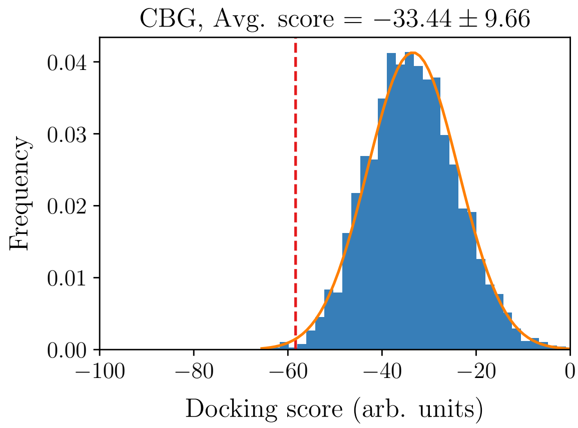

Figure S34. Histogram of docking scores for CBG

Table S32. Top 10 targets for CBG

UniProt ID	PDB ID - Chain	Protein Name	Docking Score (arb. units)	Z-Score
P45452	1FLS - A	MMP-13, Collagenase 3	-65.56	-3.99
P00797	2G21 - A	Renin	-61.81	-3.59
P43405	4I0T - A	Tyrosine-protein kinase SYK	-61.78	-3.58
P56817	2ZJJ - A	Beta-secretase 1	-61.03	-3.50
P08254	2JT5 - A	MMP-3, Stromelysin-1	-60.84	-3.48
Q9Y698	6DLZ - A	Voltage-dependent calcium channel gamma-2 subunit	-60.58	-3.45
P11309	4DTK - A	Serine/threonine-protein kinase pim-1	-60.56	-3.45
P01116	4LUC - B	GTPase KRas	-60.43	-3.44
P15056	6N0P - A	Serine/threonine-protein kinase B-raf	-60.24	-3.42
O94925	5UQE - A	Glutaminase kidney isoform, mitochondrial	-60.03	-3.39

6.7 CBGA

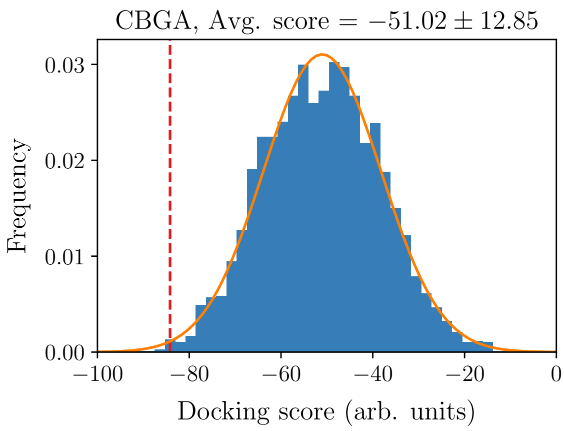

Figure S35. Histogram of docking scores for CBGA

Table S33. Top 10 targets for CBGA

UniProt ID	PDB ID - Chain	Protein Name	Docking Score (arb. units)	Z-Score
P08631	2C0T - A	Tyrosine-protein kinase HCK	-107.60	-5.25
P45452	1FLS - A	MMP-13, Collagenase 3	-97.19	-4.37
P39900	3F16 - A	MMP-12, Macrophage metalloelastase	-90.32	-3.79
O94925	5JYP - A	Glutaminase kidney isoform, mitochondrial	-88.34	-3.62
P02768	6WUW - A	Human serum albumin	-87.49	-3.55
Q9NQR1	5TH7 - B	N-lysine methyltransferase KMT5A	-86.98	-3.51
P01286	5BQM - B	Somatoliberin	-84.61	-3.30
P56817	4TRW - A	Beta-secretase 1	-83.75	-3.23
P01116	4LUC - B	GTPase KRas	-83.74	-3.23
O75530	5U8F - A	Polycomb protein EED	-83.66	-3.22

6.8 CBL

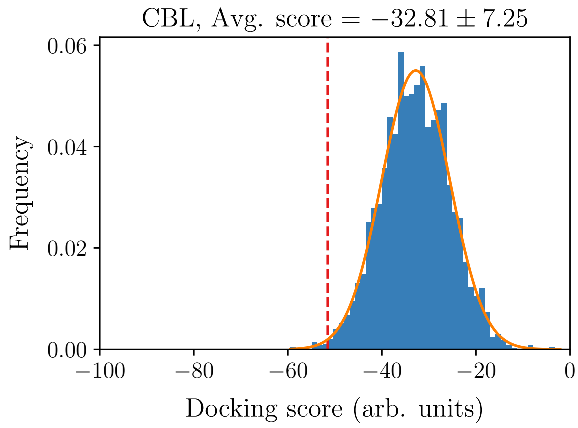

Figure S36. Histogram of docking scores for CBL

Table S34. Top 10 targets for CBL

UniProt ID	PDB ID - Chain	Protein Name	Docking Score (arb. units)	Z-Score
P08254	1SLN - A	MMP-3, Stromelysin-1	-59.51	-4.65
P45452	1FLS - A	MMP-13, Collagenase 3	-59.11	-4.58
P01116	4LV6 - B	GTPase KRas	-54.75	-3.89
Q92731	3OLL - A	Estrogen receptor beta	-54.64	-3.88
P22894	1I76 - A	MMP-8, Neutrophil collagenase	-54.60	-3.87
P14780	2OW1 - A	MMP-9, Matrix metalloproteinase-9	-54.17	-3.80
Q06187	1K2P - B	Tyrosine-protein kinase BTK	-54.03	-3.78
P56817	2VIE - A	Beta-secretase 1	-53.95	-3.77
P61088	3HCT - B	Ubiquitin-conjugating enzyme E2 N	-53.51	-3.70
P09874	4GV7 - A	Poly [ADP-ribose] polymerase 1	-52.70	-3.57

6.9 CBN

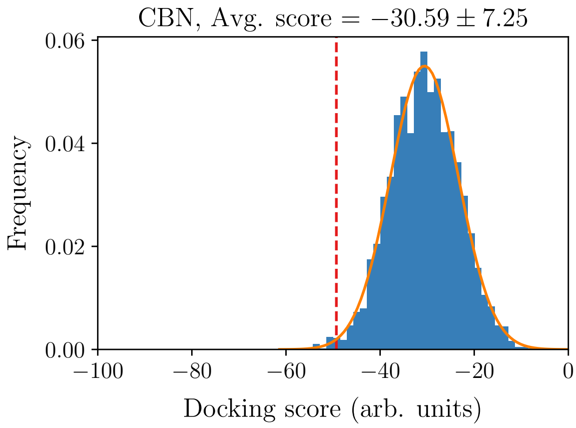

Figure S37. Histogram of docking scores for CBN

Table S35. Top 10 targets for CBN

UniProt ID	PDB ID - Chain	Protein Name	Docking Score (arb. units)	Z-Score
P08631	2C0T - A	Tyrosine-protein kinase HCK	-61.39	-5.31
P01116	4LUC - B	GTPase KRas	-57.40	-4.66
O75608	6QGN - A	Acyl-protein thioesterase 1	-53.74	-4.07
P62333	5VFT - E	26S proteasome regulatory subunit 10B	-53.16	-3.98
P12830	4ZT1 - A	Cadherin-1	-53.03	-3.96
Q16539	1WFC - A	Mitogen-activated protein kinase 14	-52.96	-3.95
P00747	1BML - A	Plasminogen	-52.86	-3.93
P08709	5PAY - C	Coagulation factor VII	-51.98	-3.79
P09467	2VT5 - B	Fructose-1,6-bisphosphatase 1	-51.14	-3.65
P39900	3F16 - A	MMP-12, Macrophage metalloelastase	-51.11	-3.65

6.10 THCA

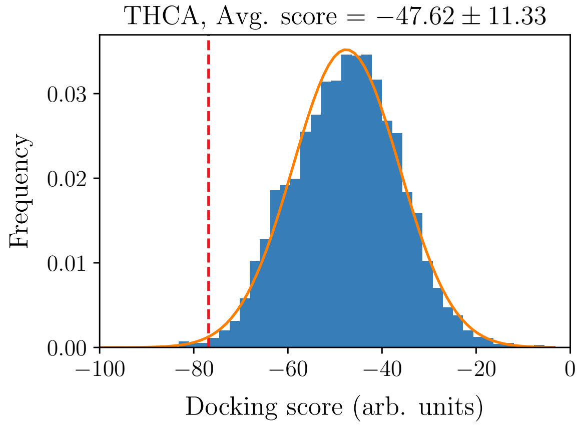

Figure S38. Histogram of docking scores for THCA

Table S36. Top 10 targets for THCA

UniProt ID	PDB ID - Chain	Protein Name	Docking Score (arb. units)	Z-Score
P08631	2C0T - A	Tyrosine-protein kinase HCK	-111.17	-6.86
P01116	4LUC - B	GTPase KRas	-89.04	-4.61
P03956	2AYK - A	MMP-1, Interstitial collagenase	-82.36	-3.93
P12830	4ZTE - A	Cadherin-1	-81.93	-3.89
P00742	2D1J - A	Coagulation factor X	-81.64	-3.86
P00746	5NAW - A	Complement factor D	-81.17	-3.81
Q07343	3WD9 - A	3',5'-cyclic-AMP phosphodiesterase 4B	-81.02	-3.80
Q9H9B1	3MO0 - A	Histone-lysine N-methyltransferase EHMT1	-80.67	-3.76
Q8IXJ6	5YQO - A	NAD-dependent protein deacetylase sirtuin-2	-80.47	-3.74
P68400	5OTH - B	Casein kinase II subunit alpha	-79.95	-3.69

6.11 THCV

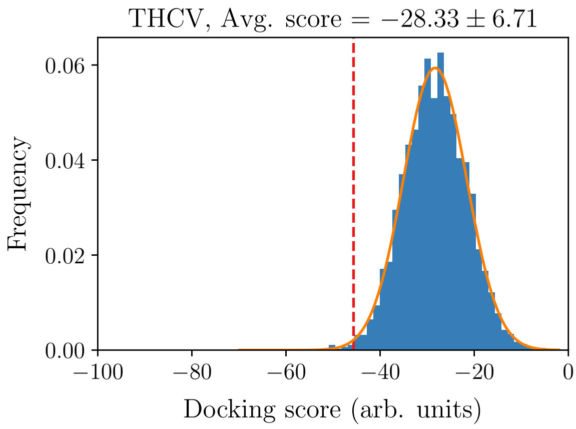

Figure S39. Histogram of docking scores for THCV

Table S37. Top 10 targets for THCV

UniProt ID	PDB ID - Chain	Protein Name	Docking Score (arb. units)	Z-Score
P08631	2C0T - A	Tyrosine-protein kinase HCK	-69.94	-7.72
P01116	4LUC - B	GTPase KRas	-59.44	-5.87
P09467	2WBB - A	Fructose-1,6-bisphosphatase 1	-51.25	-4.43
P23443	4L43 - A	Ribosomal protein S6 kinase beta-1	-50.72	-4.34
P39900	3F16 - A	MMP-12, Macrophage metalloelastase	-50.55	-4.31
P12830	4ZT1 - B	Cadherin-1	-50.43	-4.29
P00742	2D1J - A	Coagulation factor X	-49.93	-4.20
P03956	2AYK - A	MMP-1, Interstitial collagenase	-49.81	-4.18
P17787	5KXI - E	Neuronal acetylcholine receptor subunit beta-2	-48.40	-3.93
Q9NQR1	4IJ8 - A	N-lysine methyltransferase KMT5A	-47.14	-3.71

6.12 THCVA

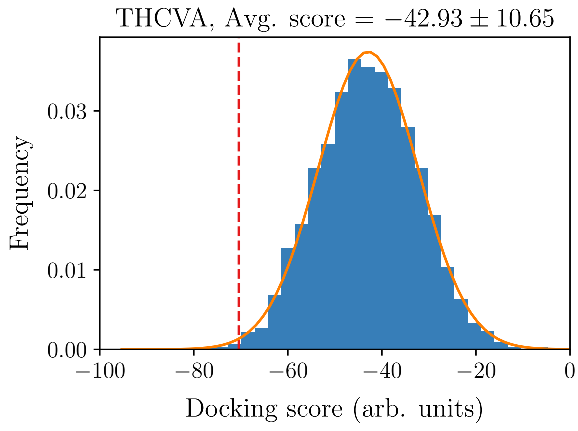

Figure S40. Histogram of docking scores for THCVA

Table S38. Top 10 targets for THCVA

UniProt ID	PDB ID - Chain	Protein Name	Docking Score (arb. units)	Z-Score
P08631	2C0T - A	Tyrosine-protein kinase HCK	-95.33	-6.18
P01116	4LUC - B	GTPase KRas	-80.52	-4.55
Q9H9B1	3MO2 - A	Histone-lysine N-methyltransferase EHMT1	-76.49	-4.10
P03956	2AYK - A	MMP-1, Interstitial collagenase	-75.30	-3.97
P00742	2D1J - A	Coagulation factor X	-73.89	-3.82
P12830	4ZT1 - A	Cadherin-1	-73.83	-3.81
P00746	5NB6 - A	Complement factor D	-72.67	-3.68
P09467	2WBD - D	Fructose-1,6-bisphosphatase 1	-72.27	-3.64
O14744	6RLL - A	Protein arginine N-methyltransferase 5	-71.69	-3.57
P28702	1H9U - D	Retinoic acid receptor RXR-beta	-71.04	-3.50

6.13 Δ-8-THC

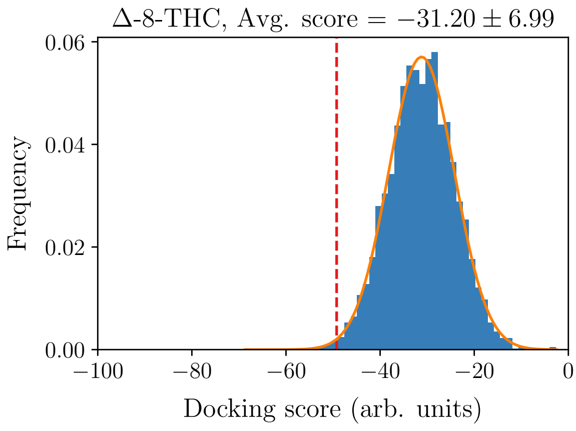

Figure S41. Histogram of docking scores for Δ-8-THC

Table S39. Top 10 targets for Δ-8-THC

UniProt ID	PDB ID - Chain	Protein Name	Docking Score (arb. units)	Z-Score
P45452	1FLS - A	MMP-13, Collagenase 3	-68.71	-6.65
P03956	2AYK - A	MMP-1, Interstitial collagenase	-58.00	-4.87
P61088	3HCT - B	Ubiquitin-conjugating enzyme E2 N	-53.48	-4.12
P11309	3VBX - A	Serine/threonine-protein kinase pim-1	-53.32	-4.09
P00742	2D1J - A	Coagulation factor X	-52.73	-3.99
P39900	3F16 - A	MMP-12, Macrophage metalloelastase	-51.80	-3.84
P02768	3LU6 - A	Human serum albumin	-51.51	-3.79
P62937	2RMB - C	Peptidyl-prolyl cis-trans isomerase A	-51.14	-3.73
P01116	4LV6 - B	GTPase KRas	-51.12	-3.72
P14780	4H82 - B	MMP-9, Matrix metalloproteinase-9	-50.30	-3.59

6.14 Δ-9-THC

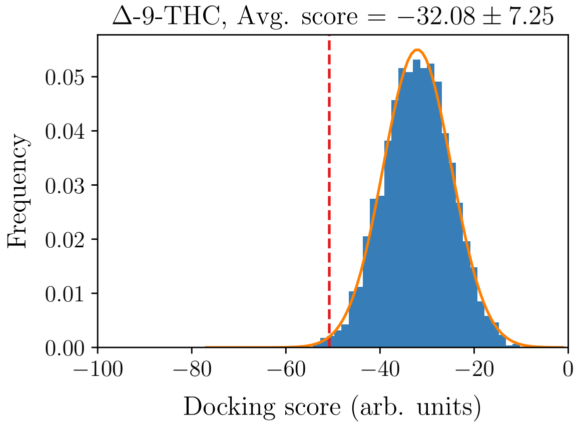

Figure S42. Histogram of docking scores for Δ-9-THC

Table S40. Top 10 targets for Δ-9-THC

UniProt ID	PDB ID - Chain	Protein Name	Docking Score (arb. units)	Z-Score
P08631	2C0T - A	Tyrosine-protein kinase HCK	-76.93	-7.72
P03956	2AYK - A	MMP-1, Interstitial collagenase	-65.31	-5.83
P39900	3F16 - A	MMP-12, Macrophage metalloelastase	-61.93	-5.28
P01116	4LUC - B	GTPase KRas	-59.54	-4.89
P12830	4ZT1 - B	Cadherin-1	-57.99	-4.63
P03372	5TLT - A	Estrogen receptor	-53.40	-3.88
P00746	5NB6 - A	Complement factor D	-53.37	-3.88
P27338	4A79 - A	Amine oxidase [flavin-containing] B	-53.21	-3.85
Q9H9B1	3MO0 - A	Histone-lysine N-methyltransferase EHMT1	-52.49	-3.74
Q6P179	5CU5 - A	Endoplasmic reticulum aminopeptidase 2	-52.41	-3.72

REFERENCES

Barratt, J. and Weitz, I. (2021). Complement factor d as a strategic target for regulating the alternative complement pathway. *Frontiers in immunology* 12, 712572

Blauvelt, A., Langley, R. G., Gordon, K. B., Silverberg, J. I., Eyerich, K., Sommer, M. O., et al. (2023). Next generation pde4 inhibitors that selectively target pde4b/d subtypes: a narrative review. *Dermatology and therapy* 13, 3031–3042

Bui, Q. T., Hong, J. H., Kwak, M., Lee, J. Y., and Lee, P. C.-W. (2021). Ubiquitin-conjugating enzymes in cancer. *Cells* 10, 1383

Camire, R. M. (2021). Blood coagulation factor x: molecular biology, inherited disease, and engineered therapeutics. *Journal of thrombosis and thrombolysis* 52, 383–390

Chelluboina, B., Nalamolu, K. R., Klopfenstein, J. D., Pinson, D. M., Wang, D. Z., Vemuganti, R., et al. (2018). Mmp-12, a promising therapeutic target for neurological diseases. *Molecular Neurobiology* 55, 1405–1409

Du, X., Song, H., Shen, N., Hua, R., and Yang, G. (2021). The molecular basis of ubiquitin-conjugating enzymes (e2s) as a potential target for cancer therapy. *International Journal of Molecular Sciences* 22, 3440

Grinshpun, A., Chen, V., Sandusky, Z. M., Fanning, S. W., and Jeselsohn, R. (2023). Esr1 activating mutations: From structure to clinical application. *Biochimica et Biophysica Acta (BBA)-Reviews on Cancer* 1878, 188830

He, J., Li, X., Zhuang, J., Han, J., Luo, G., Yang, F., et al. (2018). Blocking matrix metalloproteinase-9 abrogates collagen-induced arthritis via inhibiting dendritic cell migration. *The Journal of Immunology* 201, 3514–3523

He, X.-L., Lyu, W.-Y., Li, X.-Y., Zhao, H., Qi, L., and Lu, J.-J. (2023). Identification of glycogen phosphorylase l as a potential target for lung cancer. *Medical Oncology* 40, 211

Huang, J., Dorsey, J., Chuikov, S., Zhang, X., Jenuwein, T., Reinberg, D., et al. (2010). G9a and glp methylate lysine 373 in the tumor suppressor p53. *Journal of Biological Chemistry* 285, 9636–9641

Israelsen, W. J. and Vander Heiden, M. G. (2015). Pyruvate kinase: Function, regulation and role in cancer. In *Seminars in cell & developmental biology* (Elsevier), vol. 43, 43–51

Jin, W., Zhang, J., Chen, X., Yin, S., Yu, H., Gao, F., et al. (2023). Unraveling the complexity of histone-arginine methyltransferase carm1 in cancer: from underlying mechanisms to targeted therapeutics. *Biochimica et Biophysica Acta (BBA)-Reviews on Cancer* 1878, 188916

Kaur, R., Dahiya, L., and Kumar, M. (2017). Fructose-1, 6-bisphosphatase inhibitors: a new valid approach for management of type 2 diabetes mellitus. *European journal of medicinal chemistry* 141, 473–505

Kim, D. U., Kwak, B., and Kim, S.-W. (2019). Phosphodiesterase 4b is an effective therapeutic target in colorectal cancer. *Biochemical and biophysical research communications* 508, 825–831

Lagente, V., Le Quement, C., and Boichot, E. (2009). Macrophage metalloelastase (mmp-12) as a target for inflammatory respiratory diseases. *Expert opinion on therapeutic targets* 13, 287–295

Lerner, A., Neidhöfer, S., Reuter, S., and Matthias, T. (2018). Mmp3 is a reliable marker for disease activity, radiological monitoring, disease outcome predictability, and therapeutic response in rheumatoid arthritis. *Best Practice & Research Clinical Rheumatology* 32, 550–562

Lin, Z.-Z., Ming, D.-S., Chen, Y.-B., Zhang, J.-M., Chen, H.-H., Jiang, J.-J., et al. (2019). Kmt5a promotes metastasis of clear cell renal cell carcinoma through reducing cadherin-1 expression. *Oncology Letters* 17, 4907–4913

Lu, C., Ren, C., Yang, T., Sun, Y., Qiao, P., Wang, D., et al. (2020). A noncanonical role of fructose-1, 6-bisphosphatase 1 is essential for inhibition of notch1 in breast cancer. *Molecular Cancer Research* 18, 787–796

Luo, S., Du, S., Tao, M., Cao, J., and Cheng, P. (2023). Insights on hematopoietic cell kinase: An oncogenic player in human cancer. *Biomedicine & Pharmacotherapy* 160, 114339

Malumbres, M. (2014). Cyclin-dependent kinases. *Genome biology* 15, 1–10

Masisi, B. K., El Ansari, R., Alfarsi, L., Rakha, E. A., Green, A. R., and Craze, M. L. (2020). The role of glutaminase in cancer. *Histopathology* 76, 498–508

Milacic, M., Beavers, D., Conley, P., Gong, C., Gillespie, M., Griss, J., et al. (2024). The reactome pathway knowledgebase 2024. *Nucleic acids research* 52, D672–D678

Milite, C., Feoli, A., Horton, J. R., Rescigno, D., Cipriano, A., Pisapia, V., et al. (2019). Discovery of a novel chemotype of histone lysine methyltransferase ehmt1/2 (glp/g9a) inhibitors: rational design, synthesis, biological evaluation, and co-crystal structure. *Journal of medicinal chemistry* 62, 2666–2689

Moussa, C. E. (2017). Beta-secretase inhibitors in phase i and phase ii clinical trials for alzheimer's disease. *Expert opinion on investigational drugs* 26, 1131–1136

Nam, M.-H., Na, H., Justin Lee, C., and Yun, M. (2024). A key mediator and imaging target in alzheimer's disease: unlocking the role of reactive astrogliosis through maob. *Nuclear Medicine and Molecular Imaging* , 1–8

Nam, M.-H., Sa, M., Ju, Y. H., Park, M. G., and Lee, C. J. (2022). Revisiting the role of astrocytic maob in parkinson's disease. *International journal of molecular sciences* 23, 4453

Poh, A. R., O'Donoghue, R. J., and Ernst, M. (2015). Hematopoietic cell kinase (hck) as a therapeutic target in immune and cancer cells. *Oncotarget* 6, 15752

Pytliak, M., Vargova, V., and Mechírová, V. (2012). Matrix metalloproteinases and their role in oncogenesis: a review. *Oncology Research and Treatment* 35, 49–53

Tadesse, S., Caldon, E. C., Tilley, W., and Wang, S. (2018). Cyclin-dependent kinase 2 inhibitors in cancer therapy: an update. *Journal of medicinal chemistry* 62, 4233–4251

Tibbo, A. J. and Baillie, G. S. (2020). Phosphodiesterase 4b: master regulator of brain signaling. *Cells* 9, 1254

Timson, D. J. (2019). Fructose 1, 6-bis phosphatase: getting the message across. *Bioscience reports* 39, BSR20190124

Tursynbay, Y., Zhang, J., Li, Z., Tokay, T., Zhumadilov, Z., Wu, D., et al. (2016). Pim-1 kinase as cancer drug target: An update. *Biomedical reports* 4, 140–146

UniProtConsortium (2023). Uniprot: the universal protein knowledgebase in 2023. *Nucleic acids research* 51, D523–D531

Uprety, D. and Adjei, A. A. (2020). Kras: From undruggable to a druggable cancer target. *Cancer treatment reviews* 89, 102070

Wang, M., Sampson, E. R., Jin, H., Li, J., Ke, Q. H., Im, H.-J., et al. (2013). Mmp13 is a critical target gene during the progression of osteoarthritis. *Arthritis research & therapy* 15, 1–11

Wang, M., Zhou, Y., Huang, W., Zeng, Y., and Li, X. (2020). Association between matrix metalloproteinase-1 (mmp-1) protein level and the risk of rheumatoid arthritis and osteoarthritis: a meta-analysis. *Brazilian Journal of Medical and Biological Research* 54, e10366

Zeng, Q., He, J., Chen, X., Yuan, Q., Yin, L., Liang, Y., et al. (2024). Recent advances in hematopoietic cell kinase in cancer progression: Mechanisms and inhibitors. *Biomedicine & Pharmacotherapy* 176, 116932

Zeng, W., Zhu, J., Shan, L., Han, Z., Aerxiding, P., Quhai, A., et al. (2015). The clinicopathological significance of cdh1 in gastric cancer: a meta-analysis and systematic review. *Drug design, development and therapy* , 2149–2157

Zhao, Y., Aziz, A. u. R., Zhang, H., Zhang, Z., Li, N., and Liu, B. (2022). A systematic review on active sites and functions of pim-1 protein. *Human Cell* , 1–14

Zheng, Y., Liu, A., Wang, Z.-J., Cao, Q., Wang, W., Lin, L., et al. (2019). Inhibition of ehmt1/2 rescues synaptic and cognitive functions for alzheimer's disease. *Brain* 142, 787–807

Zois, C. E., Hendriks, A. M., Haider, S., Pires, E., Bridges, E., Kalamida, D., et al. (2022). Liver glycogen phosphorylase is upregulated in glioblastoma and provides a metabolic vulnerability to high dose radiation. *Cell death & disease* 13, 573
